# A Shiga Toxin B-Subunit-Based Lectibody Boosts T Cell Cytotoxicity towards Gb3-Positive Cancer Cells

**DOI:** 10.3390/cells12141896

**Published:** 2023-07-20

**Authors:** Jana Tomisch, Vincent Busse, Francesca Rosato, Olga N. Makshakova, Pavel Salavei, Anna-Sophia Kittel, Emilie Gillon, Levin Lataster, Anne Imberty, Ana Valeria Meléndez, Winfried Römer

**Affiliations:** 1 Faculty of Biology, University of Freiburg, 79104 Freiburg, Germany; 2 Signalling Research Centres BIOSS and CIBSS, University of Freiburg, 79104 Freiburg, Germany; 3 Kazan Institute for Biochemistry and Biophysics, FRC Kazan Scientific Center of RAS, 420111 Kazan, Russia; 4 Core Facility Signalling Factory & Robotics, University of Freiburg, 79104 Freiburg, Germany; 5 CNRS, CERMAV, Université Grenoble Alpes, 38000 Grenoble, France; 6 Spemann Graduate School of Biology and Medicine, University of Freiburg, 79104 Freiburg, Germany; 7 Freiburg Institute for Advanced Studies (FRIAS), University of Freiburg, 79106 Freiburg, Germany

**Keywords:** bispecific T cell engager, lectins, Shiga toxin B-subunit, cancer immunotherapy, tumour-associated carbohydrate antigens, globotriaosylceramide, T cells, glycans, glycosphingolipids, solid tumours, colon cancer, Burkitt’s lymphoma

## Abstract

Aberrant glycosylation plays a crucial role in tumour progression and invasiveness. Tumour-associated carbohydrate antigens (TACAs) represent a valuable set of targets for immunotherapeutic approaches. The poor immunogenicity of glycan structures, however, requires a more effective and well-directed way of targeting TACAs on the surface of cancer cells than antibodies. The glycosphingolipid globotriaosylceramide (Gb3) is a well-established TACA present in a multitude of cancer types. Its overexpression has been linked to metastasis, invasiveness, and multidrug resistance. In the present study, we propose to use a dimeric fragment of the Shiga toxin B-subunit (StxB) to selectively target Gb3-positive cancer cells in a StxB-scFv UCHT1 lectibody. The lectibody, comprised of a lectin and the UCHT1 antibody fragment, was produced in *E. coli* and purified via Ni-NTA affinity chromatography. Specificity of the lectibody towards Gb3-positive cancer cell lines and specificity towards the CD3 receptor on T cells, was assessed using flow cytometry. We evaluated the efficacy of the lectibody in redirecting T cell cytotoxicity towards Gb3-overexpressing cancer cells in luciferase-based cytotoxicity in vitro assays. The StxB-scFv UCHT1 lectibody has proven specific for Gb3 and could induce the killing of up to 80% of Gb3-overexpressing cancer cells in haemorrhagic and solid tumours. The lectibody developed in this study, therefore, highlights the potential that lectibodies and lectins in general have for usage in immunotherapeutic approaches to boost the efficacy of established cancer treatments.

## 1. Introduction

Cancer is one of the leading causes of death worldwide. In 2020, ten million people died of cancer, and increasing death counts are to be expected each year [1]. Conventional treatment methods, such as surgery, chemotherapy, or radiotherapy are viable options. Yet, they cannot be exclusively guided towards cancer eradication and tend to have serious side effects on the human body in the form of physical pain, a weakened immune system, or even the resurgence of tumours [2,3,4,5,6]. It is necessary, thus, to find more selective approaches to combat cancer. Immunotherapy has shown new and encouraging paths for treating cancer in recent years [7,8]. Administering customised antibody treatments or vaccinations into the patient’s body succeeded as cancer cells have been targeted more effectively and, most importantly, with less severe outcomes for healthy cells and tissues [9].

One of the first breakthroughs of immunotherapy was the generation of monoclonal antibodies (mAbs) exclusively targeting one antigen. The first mAbs were created by fusing monoclonal B cells with myeloma cells [10]. Over the decades, this revolutionary concept was improved upon in several ways. mAbs were fused via linkage to form bispecific antibodies (bsAbs), enabling the targeting of two cells with differing epitopes simultaneously [11]. Further advances in genetic engineering allowed the use of the minimal binding domains of mAbs, coupling only their light chain and the heavy chain to form the so-called single-chain variable fragment (scFv) [12,13]. The scFvs demonstrated better tumour penetration due to their reduced molecular weight, absence of glycosylation, and an anti-tumour activity independent of the Fc region [14]. They could, additionally, be produced in a less expensive and more efficient process than conventional mAbs [15]. Unique chimeric fusion proteins emerged, a major class of them being the bispecific T cell engagers (BiTEs). Typically, BiTEs consist of two linker-fused scFvs, having an αCD3 antibody fragment aimed towards CD3 of the T cell receptor (TCR) and a second scFv aimed against antigens exposed on the cancer cell surface [11,16,17]. The linker between the two scFvs enables a flexible orientation of both parts towards their targets and allows the closer proximity of T cells and target cells, resembling an immunological synapse [16,18,19]. By these means, BiTEs bind to their intended targets while simultaneously activating T cells and triggering their cytotoxic signalling. The activation of cytotoxic T cells via the CD3 receptor is independent of the antigen recognition from the major histocompatibility complex I (MHC I) and in the absence of co-stimulatory molecules [20,21]. Upon activation, among other mechanisms, T cells release perforin, which creates pores in the target cell membrane, enabling the passage of granzymes into the cytoplasm, ultimately inducing apoptosis in the cancer cells [22,23,24]. Other mechanisms following after the activation of T cells include the proliferation, expansion, and differentiation of T cells, and cytokine production [25,26]. The first BiTE approved by the Food and Drug Administration (FDA) was blinatumomab, which targets the CD19 surface antigen in B cell acute lymphoblastic leukaemia [27,28]. Several other BiTEs containing αCD3 are in differing stages of clinical trials, such as the Siglec-3 (CD33)-binding AMG 673 directed against relapsed/refractory acute myeloid leukaemia, the PSMA-targeting pasotuxizumab in prostate cancer, or the BiTE AMG 596, which is aimed towards EGFRvIII on glioblastoma [29,30,31,32]. 

Cancer development is often accompanied by aberrant glycosylation leading to changes in the glycan structure of normal cells, benefiting tumour development and enhancing cancer cell functionality [33]. Altered glycosylation is commonly reflected in the overexpression of surface antigens, so-called tumour-associated carbohydrate antigens (TACAs). Truncated and neoantigens are also part of TACAs. These aberrant structures are key regulators of cancer proliferation and invasiveness, and the formation of metastasis [34,35]. Glycosphingolipids (GSLs) are of increased interest in cancer therapies [36]. They are mostly located on the outer plasma membrane leaflet and, to some extent, undergo site-specific, protein-specific, or cell-specific alterations favouring cancerogenic development. The globo-series represents a major class of GSLs driving tumour progression and sustaining cancer metabolism [37]. Their presence on cell surfaces provides a potential set of targets for the development of new immunotherapeutic strategies. Among the GSL subclass of globosides, one relevant intermediate is globotriaosylceramide (Gb3). Gb3 is composed of a ceramide backbone and a neutral trisaccharide; the ceramide contains sphingosine and a fatty acyl chain with variations in its length, hydroxylation, and saturation degree [38,39]. The trisaccharide (comprised of two galactose (Gal) molecules and one glucose (Glc) molecule) is added onto the ceramide, forming Gb3 (αGal1-4βGal1-4βGlc1-Cer) [40]. The addition of the sugar molecules is initiated by the linkage of galactose onto a lactosylceramide backbone by the α1,4-galactosyltransferase, also referred to as the Gb3 synthase [41,42]. Gb3 is present in erythrocytes as the P^k^ blood group antigen and in the germinal centre of B cells as the CD77 receptor [43,44]. Gb3 was verified in various cancer types, such as Burkitt´s lymphoma, colon, gastric, breast, pancreatic, and ovarian cancer [45,46,47,48,49,50]. The definitive function of Gb3 in tumours has not yet been determined. It is anticipated to be involved in cancer survival such as metastasis (in colon cancer) or multidrug resistance (in breast cancer, colon cancer, ovarian cancer) [46,51,52]. The sum of all these features turns Gb3 into an immunotherapeutic target of increased interest.

On the downside, TACAs, including Gb3, are poorly immunogenic [53,54]. In combination with the antigen-abolishing properties of some tumours, the purpose of targeting Gb3 via conventional antibodies is defeated [55,56]. However, a group of carbohydrate-binding proteins, namely lectins, can bind Gb3 independently from its innate immunogenicity. One of these is the *Shigella dysenteriae*-originated Shiga Toxin (Stx), discovered by Dr. Kiyoshi Shiga in 1897 [57]. Stx has also been detected in specific strains of *Escherichia coli*, thus being called Shiga toxin-producing *E. coli* [58]. Other noteworthy lectins with Gb3 specificity are LecA, originating from *Pseudomonas aeruginosa*, SadP from *Streptococcus suis,* and the artificially engineered Mitsuba, which stems from the mussel (*Mytilus galloprovincialis*) lectin MytiLec [40,59,60]. Stx is composed of two subunits, each with a particular task. The Shiga toxin A subunit (StxA) is enzymatically active and possesses *N*-glycosidase activity, which inhibits protein synthesis via the modification of host ribosomal RNA [61,62]. On the other hand, the non-toxic homopentameric Shiga toxin B-subunit (StxB) binds Gb3 specifically and multivalently. Each monomer of StxB possesses three ligand-binding sites, offering up to 15 binding opportunities for its target [61]. Upon binding Gb3, StxB can induce the formation of clusters that lead to negative membrane curvature and invagination, accompanying retrograde transport into the cell [61,63,64,65]. Interestingly, StxB is also fully functional in the absence of StxA [66].

Together, these aspects turn StxB into an engaging tool for addressing cancers that are characterized by the presence of Gb3 on their cell surfaces [45,46,47,48,49,50]. StxB has already been explored as a tool in cancer therapy, with promising results further encouraging its use in cancer treatment [49,50,67,68,69,70]. We envisage tackling this issue by relying on the BiTE concept, replacing the TACA-targeting scFv with a monomeric StxB and fusing the lectin to the scFv of an αCD3 antibody. Our approach might help to circumvent the immune escape mechanisms of tumours by opening attractive possibilities for targeting glycans to ultimately eliminate cancer cells, which is preceded by the activation of cytotoxic T cells in an MHC I-independent manner. It is highly encouraging that a fusion protein of monomeric StxB and a truncated diphtheria toxin, containing solely its catalytic A subunit, has already been established and tested extensively, showing stable expression and binding of monomeric StxB to human breast adenocarcinoma cell lines in the fusion protein [71].

## 2. Materials and Methods

### 2.1. Antibodies and Chemical Reagents

The following antibodies were purchased from BioLegend (San Diego, CA, USA): Alexa Fluor^TM^ 647 αHisTag Antibody (Cat. No.: 652513) and Purified anti-HisTag Antibody (Cat. No.: 652501). Peroxidase AffiniPure Donkey αMouse IgG (H + L) antibody (Cat. No.: 715-035-151) was bought from Jackson ImmunoResearch Laboratories, Inc. (West Grove, PA, USA).

Roswell Park Memorial Institute 1640 medium (RPMI 1640), fetal bovine serum (FBS), L-Glutamine, HEPES, and 0.05% Trypsin-EDTA (1×) were commercially bought from Thermo Fisher Scientific (Waltham, MA, USA). DMSO, Isopropyl-β-D-thiogalactopyranoside (IPTG), penicillin/streptomycin, ß-mercaptoethanol, ampicillin, and lysogeny broth (LB)-medium were purchased from Carl Roth GmbH (Karlsruhe, Germany). PBS, Pancoll, and Minimum Essential Medium (MEM) Non-Essential Amino Acid Solution (100×) were obtained from PAN Biotech (Aidenbach, Germany). Dulbecco´s Modified Eagle´s Medium (DMEM) was provided by Capricorn Scientific GmbH (Ebsdorfergrund, Germany). The DMEM was already supplied with 1.0 g/L L-glucose, 1.0 g/L L-Glutamine, 1.0 g/L sodium pyruvate, and 3.7 g/L NaHCO_3_ D-Luciferin Firefly was obtained from Biosynth (Staad, Switzerland).

### 2.2. Cell Lines

In this study, Burkitt´s lymphoma-derived Ramos and Namalwa cell lines, and HT29 and LS174 colon adenocarcinoma cell lines were used (Ramos cells were kindly provided by Prof. Dr. Michael Reth, Institut für Biologie III, Albert-Ludwigs Universität Freiburg, Germany; Namalwa.CSN/70, ACC 70, DSMZ—German Collection of Microorganisms and Cell Cultures GmbH; HT29 and LS174 cells were kindly provided by Prof. Dr. Susana Minguet, Institut für Biologie III, Albert-Ludwigs Universität Freiburg, Germany).

Ramos and Namalwa cells were maintained in RPMI 1640 medium containing 10% heat-inactivated FBS, 5 µg/mL penicillin/streptomycin (P/S), and 2 mM L-Glutamine. Human colon adenocarcinoma cell lines HT29 and LS174 were kept in DMEM supplied with, 10% FBS, 2.5 µg/mL P/S, 1% HEPES, and 5 mL of MEM Non-Essential Amino Acid solution. All cell lines were maintained at 37 °C and 5% CO_2_.

### 2.3. Isolation of PBMCs

A density gradient was used for isolating peripheral blood mononuclear cells (PBMCs), blood from healthy donors was mixed 1:1 with PBS. The solution was then placed on top of 20 mL Pancoll in a centrifuge tube. The blood-PBS solution was added dropwise to form single pearl-like structures. The tubes were spun down for 30 min with 141× *g* at RT. After spinning, the PBMCs were collected from the gradient (plasma was on top of the gradient, PBMCs were located directly below, present in a thin layer) and washed with PBS, and subsequently spun down for 10 min at 398× *g*. The washing step was repeated once. If red blood cells were present in the cell pellet, 1 mL ammonium–chloride–potassium (ACK) lysis buffer was added to the cells and incubated for 4 min. Later, the cells were pelleted by the last centrifugation step for 5 min at 141× *g* and the lysis buffer was removed. In total, 2 × 10^7^ cells were frozen in a single cryovial and stored at −80 °C. After thawing, the PBMCs were cultivated in RPMI 1640 medium with 10% FBS, 2.5 µg/mL P/S, 1% HEPES and 0.0035% β-Mercaptoethanol at 37 °C and 5% CO_2_.

### 2.4. Homology Modeling of the Lectibody Structure

The structure of the chimeric StxB-scFv UCHT1 fusion protein was built up using Modeller9.15 (San Francisco, CA, USA) [72]. Diabody 31 (PDB code: 6KR0) was used as a template for the αCD3 scFv (the relative alignment is given in Supplementary Figure S1). The StxB monomer structure was derived from the X-ray structure of StxB pentamer (PDB code: 1BOS) 

### 2.5. Protein–Protein Docking for Monomeric Lectibody Design

The structure of StxB monomer and homology-based structure of scFv were used for protein–protein docking. The docking was performed using ClusPro web server [73,74]. The top thirty docking poses were taken for the analysis. 

### 2.6. Structure Optimization

The spatial structure of the whole fusion protein was built up retaining the StxB and scFv mutual orientation selected from docking results. The linker was added using Modeller9.15 (San Francisco, CA, USA). The structure was equilibrated using molecular dynamics (MD). MD simulations were carried out in the Amber18 [75] using the Amber14SP force-field parameters. Fusion protein was immersed into a water box with periodic boundary conditions. The TIP3P model was used for water molecules. Sodium and chloride ions were added in the amount necessary to neutralize the protein’s charge and to maintain the ionic strength of 150 mM. The integration step of 2 fs was used together with the SHAKE algorithm constraining the bonds involving hydrogen atoms [76]. The Particle mesh Ewald (PME) method was used for long-ranged electrostatic interactions [77]. The simulations were carried out in the isotherm isobar thermodynamic ensemble at 300 K. The temperature and the pressure were kept constant using a Langevin thermostat with a collision frequency of 2 ps^−1^ and a weak coupling algorithm with a relaxation time of 2 ps, respectively. First, the system was minimized for 5000 steps and then equilibrated. In the production run, 1000 ns of the trajectory were accumulated. The trajectory analysis (including per-residue contacts and interaction energy) was performed on the last 200 ns.

### 2.7. Transformation of the StxB-scFv UCHT1 Lectibody Sequence

The sequence of StxB-scFv UCHT1 was custom-ordered in a pET22b+ plasmid (BioCat GmbH, Heidelberg, Germany). The chemically competent BL21(DE3) *E. coli* strain (New England Biolabs, Ipswich, MA, USA) was used for transformation, following the manufacturer´s instructions. The bacteria were briefly thawed on ice, and then 10 ng of DNA was added. The mix was incubated for 30 min at 4 °C. Afterwards, the reaction was stopped by heat shocking the bacterial mix for 20 s at 42 °C. Following this step, 350 µL of sterile LB-medium was added and incubated at 37 °C for 1 h under agitation (180 rpm). The solution was streaked out on LB agar plates containing ampicillin. The plates were kept overnight at 37 °C.

Following the incubation time, a single colony was picked and transferred to a tube with LB-medium. The culture grew overnight at 37 °C under continuous shaking (180 rpm). Afterwards, 100 mL of LB-medium was inoculated with the overnight culture, in the same conditions as mentioned above. The plasmid DNA of the bacteria was then purified (NucleoBond PC 500 kit, Macherey Nagel, Düren, Germany). The plasmid map of the resulting plasmid can be seen in Supplementary Figure S2.

### 2.8. Protein Expression and Purification

A preculture was started with transformed bacteria and transferred to 60 mL LB-medium. The preculture was incubated overnight at 37 °C under continuous shaking (180 rpm). Then, three litres of LB-medium was inoculated with the preculture and ampicillin was added to a final concentration of 100 µg/mL. The flasks were shaken with 180 rpm at 37 °C, their optical density (OD) was measured regularly. As soon as an OD of 0.6 was attained, IPTG was added to a final concentration of 0.5 mM and the temperature was reduced to 20 °C. The culture was shaken for 16 h at 140 rpm. Following this, the bacterial solution was collected and centrifuged for 15 min at 4 °C and 4790× *g*. The supernatant was discarded, and the pellets resuspended in binding buffer (25 mL buffer/1 L culture volume; 20 mM NaH_2_PO_4_, 500 mM NaCl, 20 mM Imidazole, pH 8.0). The cell walls were disrupted with a CF1 high-pressure homogenizer, French Press, and an applied pressure of 2 kbar in two passages. After disruption, cells were centrifuged once more for 30 min at 4 °C and 30,000× *g*. Next, the supernatant was recovered and purified via HisTag affinity chromatography (elution buffer: 20 mM NaH_2_PO_4_, 500 mM NaCl, 500 mM Imidazole, pH 8.0). A HiLoadTM 26/600 SuperdexTM 200 pg column (Sigma-Aldrich, St. Louis, MO, USA) was used in an ÄKTA avant chromatography system (Cytiva, Marlborough, MA, USA). Immediately following the purification, the purified proteins were run in a size exclusion chromatography (SEC) in the device mentioned before.

### 2.9. Sodium Dodecyl Sulphate Polyacrylamide Gel Electrophoresis (SDS-PAGE) and Immunoblotting

To confirm the calculated protein size, an SDS-PAGE gel electrophoresis followed by immunoblotting was prepared. To this end, non-reducing sample buffer (4×) (2 mL MilliQ water, 0.8 g SDS, 2 mL 0.5 M Trizma-base, 0.4% bromophenol, 4 mL glycerol, filled to 10 mL with ddH_2_O) was added to 10 µg protein samples and boiled for 5 min at 95 °C. A 12% acrylamide gel was loaded with the samples and run in a PowerPac™ Basic Power Supply (Bio-Rad Laboratories, Hercules, CA, USA) for 20 min at 100 V, following another running time of 45 min at 120 V. The proteins were subsequently transferred to a nitrocellulose membrane with a current of 0.19 mA per gel, for 30 min (peqPOWER 250 Volt Power Supply, VWR International, Radnor, PA, USA). Then, the membrane was incubated for 30 min at RT with blocking solution (3% BSA in TBS-T). Afterwards, the membrane was incubated with an anti-6x-HisTag antibody (1:1000, BioLegend, San Diego, CA, USA) overnight at 4 °C under continuous shaking. All antibodies were diluted in blocking solution. On the next day, the membrane was washed three times with TBS-T for 5 min each. Corresponding horseradish peroxidase-conjugated secondary antibody (1:1000, Jackson ImmunoResearch Laboratories, West Grove, PA, USA) was added to the membrane for 1 h at RT. After incubation, the membrane was washed. Finally, luminescence was detected using a Novex^®^ Electrochemiluminescence kit (Invitrogen AG, Carlsbad, CA, USA). Images were acquired using a FUSION FX imager and the FusionCapt Advance Solo software V.17.04a (both Vilber Lourmat Deutschland GmbH, Eberhardzell, Germany).

### 2.10. Flow Cytometry

To assess the binding of the lectibody to target cells, 2 × 10^5^ tumour cells were plated in a 96-well U-bottom plate. The cells were spun down for 3 min at 1600× *g* and 4 °C. All following centrifugation procedures were performed with these settings. The cells were washed once with 200 µL of FACS buffer (3% FBS in PBS, *v*/*v*) and spun down. All washing steps were performed with FACS buffer. The target cells were resuspended in fresh medium containing StxB-scFv UCHT1 in three differing concentrations (14, 69, 138 nM) and incubated for 30 min at 4 °C. Afterwards, the cells were centrifuged and washed once with FACS buffer, and then the antibody Alexa Fluor^TM^ 647 anti-6x-HisTag (Biolegend, San Diego, CA, USA) was added for 15 min at 4 °C in a dilution of 1:1000. Following this procedure, the cells were washed three times in total and then analysed with the Gallios Flow Cytometer (Beckman Coulter Inc., Brea, CA, USA). The data were processed with the FlowJo software (V. 10.05.3; Becton Dickinson, Franklin Lakes, NJ, USA).

### 2.11. Cytotoxicity Assay

To determine the capability of the lectibody in mediating cross-linking of target and effector cells and inducing target cells lysis, 1 × 10^4^ tumour cells (Ramos, Namalwa, HT29 and LS174 transfected with the gene for luciferase and GFP reporter synthesis) were each plated in triplicate one day prior to experiment in a 96-well flat-bottomed plate. On the next day, the medium was exchanged with new culture medium containing 75 µg/mL luciferin. PBMCs were added to the target cells in a 5:1 effector to target (E:T) ratio, and StxB-scFv UCHT1 was added in varying concentrations (14, 69, 138 nM). The plate was incubated at 37 °C, 5% CO_2_, and bioluminescence (BLI) was measured at 7, 24, and 48 h. BLI of the cells was measured as relative light units (RLUs) in a luminometer (Tecan Infinity M200 Pro, Tecan, Männedorf, Switzerland). Target cells co-incubated with PBMCs lacking lectibody indicated the baseline cell death, while target cells incubated solely with 2% Triton X-100 showed maximal cell death. The percentage of specific killing was calculated by using the following formula:

$$specific\;killing\;activity=\frac{\mathrm{RLU}\;\left(\mathrm{average}\;\mathrm{spontaneous}\;\mathrm{cell}\;\mathrm{death}\right)-\;\mathrm{RLU}\;\left(\mathrm{measurement}\right)}{\mathrm{RLU}\;\left(\mathrm{average}\;\mathrm{spontaneous}\;\mathrm{cell}\;\mathrm{death}\right)-\;\mathrm{RLU}\;\left(\mathrm{average}\;\mathrm{maximal}\;\mathrm{cell}\;\mathrm{death}\right)}\times 100$$

The measured data were analysed using GraphPad Prism (V. 8.4.3; GraphPad Software Inc., San Diego, CA, USA). 

### 2.12. Cell Proliferation (MTT) Assay

To investigate T-cell-independent cytotoxicity of StxB-scFv UCHT1 on human cells, Ramos, Namalwa, HT29, and LS174 cells were treated with increasing concentrations of lectibody for 24 h in a standard MTT assay. A total of 3 × 10^4^ cells per well were transferred to a 96-well U-bottom plate. The cells were centrifuged at 1600× *g* for 3 min at RT. The cell pellet was resuspended in 100 μL of variously concentrated protein solutions (14, 69, 138 nM) and transferred to a 96-well flat-bottomed plate. The cells were incubated for 24 h at 37 °C. Subsequently, 10 μL of MTT labelling solution (MTT Cell Proliferation Kit, Roche Holding, Basel, Switzerland) was added to each well, and the cells were incubated for 4 h at 37 °C. Then, 100 μL of the solubilisation reagent was added to each well, and the plate was stored for incubation at 37 °C overnight. Metabolically active cells can take up the yellow tetrazolium salt MTT and reduce it to purple formazan, which is then exocytosed. After solubilisation, the amount of formazan in the medium can be measured by its absorbance. The next day, the absorbance of the samples was measured at 550 nm using a BioTek microplate reader (BioTek Instruments, Inc., Winooski, VT, USA). The data were further analysed using GraphPad Prism software (V. 8.4.3; GraphPad Software Inc., San Diego, CA, USA).

### 2.13. Affinity Measurement Using Surface Plasmon Resonance (SPR)

All experiments were performed on a BIACORE X100 (Cytivia, Marlborough, MA, USA) at 25 °C. For activation and immobilization of the lectibody, buffer A (20 mM Na_2_HPO_4_ pH 9.4 + NaCl 500 mM + 0.05% Tween 20) was used as a running buffer. Two CM5 BIACORE chips were activated by 2 injections of 1:1 NHS/EDC mixture (contact time = 600 s, flow rate = 5 µL/min) on channel 1 and 2, followed by injections of the lectibody (monomeric lectibody: 226 µg/mL in 10 mM sodium acetate buffer pH 4.5 on channel 2 only until the final binding response reached 3800 RU; dimeric lectibody: same conditions until immobilization of 5877 RU). The interaction with Gb3 was investigated by injection at different concentrations (5000 µM, 1667 µM, 555.5 µM, 185.2 µM) on the chips (contact time = 200 s, dissociation time = 200 s, flow rate 20 µL/min).

### 2.14. Statistical Analysis

To evaluate statistical relevance, an ordinary one-way ANOVA was performed comparing the mean of each treatment to the mean of the control. The data were corrected for multiple comparison using Holm–Sidak test. The significance was determined when *p* < 0.05 and the confidence level was set to the 95% confidence interval. The statistical analysis was performed using GraphPad Prism (V. 8.4.3; GraphPad Software Inc., San Diego, CA, USA).

## 3. Results

### 3.1. Design and Structural Prediction of the StxB-scFv UCHT1 Lectibody

**Figure 1:**
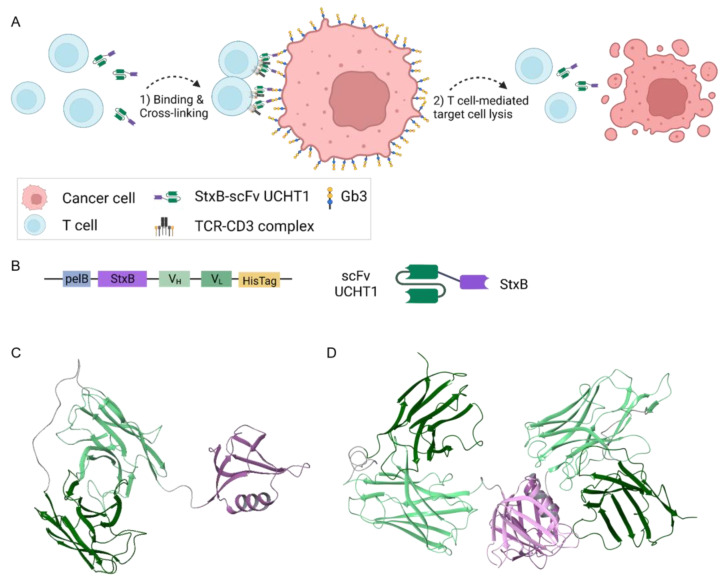
Schematic representation of cancer cell killing by activated T cells via StxB-scFv UCHT1 lectibody-mediated cross-linking, lectibody sequence, and atomic models. (**A**) When incubated together, the lectibody connects T cells (blue) with cancer cells (red). Created with BioRender.com. (**B**) One monomer fragment of StxB is connected to the scFv UCHT1 via a short (1× GGGS) linker. The heavy chain (V_H_) of the antibody fragment is connected to the light chain (V_L_) via a long linker (4× GGGS). (**C**) A homology-based model of a monomeric StxB-scFv UCHT1 lectibody. (**D**) A homology-based model of a dimeric StxB-UCHT1 lectibody. Colour coding: StxB monomer fragment—violet, heavy chain variable fragment—light green, light chain variable fragment—dark green, linkers—dark grey.

The lectibody design was inspired by the BiTEs, which combine a target antigen-binding antibody with an αCD3 antibody as one recombinant molecule. BiTEs bind to CD3 in a monovalent fashion with low affinity, and only trigger T cell signalling when multiple molecules bind simultaneously and therefore cluster the CD3 receptors [78]. Cytotoxic T cells are activated via CD3 receptor clustering accompanied by conformational changes [79]. Since the engineered lectibody follows the design of BiTEs, it hypothetically cross-links a T cell (Figure 1A, blue) to a cancer cell (Figure 1A, red) and in turn activates the T cell by clustering the CD3 receptor leading to cancer cell apoptosis. The StxB-scFv UCHT1 lectibody was designed as a recombinant fusion protein. To this end, one subunit of StxB was connected over a linker to the heavy (V_H_) and light (V_L_) chain of a scFv UCHT1 αCD3 antibody and fused to a 6x-HisTag at the C-terminus (Figure 1B and Supplementary Figure S3). 

The stability of the pentameric StxB is strongly determined by the inter-subunit interactions [80]. Outside of the pentamer, the stability of the StxB monomer fragment can be improved by fusing it with other protein modules [71]. Here we attempted to design a monomeric StxB–scFv UCHT1 fusion protein, assuming that the unfavourable exposure of StxB hydrophobic groups to water may be hidden via direct interactions of the scFv with StxB in the region of its inter-subunit contacts. Such interactions could prevent pentamer formation and stabilize the fold of the monomer fragment. 

To explore alternative interactions between StxB and scFv UCHT1 and to estimate the length of the linker required to connect the two protein modules, we performed a protein–protein docking using ClusPro web server. The docking revealed the tendency of the scFv UCHT1 to interact with StxB using the Gb3-binding surface of the latter (Supplementary Figure S4A). In such complexes, the distance between the C-terminus of StxB and the N-terminus of scFv amounts to about 30 Å. Thus, one may conclude that, in the functional fusion protein, the linker connecting StxB and scFv should be shorter than 30 Å in order to prevent the blockage of Gb3 binding because of direct protein contacts. 

The desired interactions between StxB and scFv monomers in the hydrophobic regions, which are involved in the pentamer formation, are also probable. Two docking poses meeting this condition are shown in Supplementary Figure S4B,C. In these complexes, the distance between the C-terminus of StxB and the N-terminus of scFv is less than 12 Å. Therefore, to increase the probability of StxB and scFv to form such hydrophobic contacts and impede the pentamer formation, for the fusion protein, we chose a flexible linker of ~12 Å in length. 

As the next step of modelling, the full structure of the fusion protein, i.e., StxB and scFv monomers connected via the GGGS linker, was equilibrated using MD. In the two starting conformations used for MD simulations, the mutual orientations of StxB and scFv monomers were kept the same as those revealed by docking. These conformations were refined in the course of two 1 µs MD trajectories with explicit water. The root-mean-square deviation analysis revealed that in the course of trajectories, the scFv module of the lectibody explored the space to adopt a favourable orientation in respect of the StxB module (see Supplementary Figure S5). The per-residue contact analysis in the equilibrated structures of the lectibody (see Supplementary Table S1) showed that two trajectories merged over time, resulting in one stable conformation depicted in Supplementary Figure S6. In the equilibrated structure, the scFv interacts with the residues of StxB (Arg33, Trp34, Asn35, Gln37, Ser38, Leu39, Leu41, Ser42, Ile45, Thr46, Met48, Ile67) that are involved in the pentamer formation in native StxB folding [81]. Importantly, the folding of both the StxB and scFv domains of the lectibody was revealed as stable. 

Summing up, the modelling suggested that the GGGS short linker, connecting the StxB and the αCD3 fragment, prevents potential blockage of the Gb3-binding sites as a result of direct protein–protein contacts. The evolution of protein–protein contacts during MD trajectories revealed the tendency of the lectibody to keep a compact organization. In the equilibrated conformation, the mutual orientation of StxB and the αCD3 fragment creates a steric conflict hindering the StxB oligomerization (Supplementary Figure S6). Interestingly, the monomeric StxB seems to be stable when attached to the scFv αCD3, in agreement with the DT389-STXB fusion construct developed by Mohseni and colleagues [71]. Further analysis revealed a capability of the monomeric StxB-scFv UCHT1 fusion protein to form a dimer (Figure 1D). Due to its large size, one scFv UCHT1 can interact with two StxB subunits in their hydrophobic face, the other scFv UCHT1 remains flexible (Supplementary Figure S5, Supplementary Table S1).

### 3.2. Production and Selection According to Size of the StxB-scFv UCHT1 Lectibody

**Figure 2:**
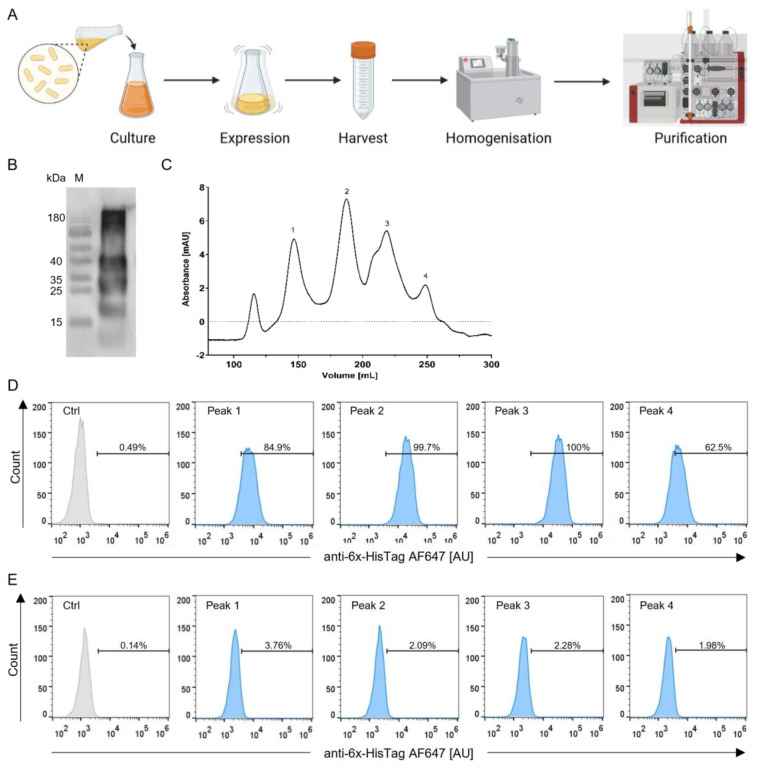
Production, selection, and binding profile of the StxB-scFv UCHT1 lectibody. (**A**) Six litres of LB-medium were inoculated with 120 mL of precultured *E. coli* BL21(DE3) carrying the StxB-scFv UCHT1 lectibody plasmid. After the cultures reached an OD of 0.6, protein expression was induced with 0.5 mM IPTG. The protein was expressed overnight and then harvested by centrifugation. The pellet was then homogenised using a French press. Afterwards, the lysate was filtered and purified via Ni-NTA affinity purification followed by SEC. Created with BioRender.com. (**B**) SDS-PAGE showing different bands of the purified protein. As the affinity purification resulted in a mixture of protein sizes, SEC was performed. (**C**) Exemplary size exclusion chromatography graph. Protein elution is displayed in relation to UV absorbance in milli-Absorbance Units (mAU) to volume in mL. The size of the protein corresponds to the elution volume. (**D**) Ramos (high abundance of Gb3) and (**E**) Namalwa cells (low abundance of Gb3) were incubated with 14 nM of lectibody derived from each peak to assess StxB-scFv UCHT1 binding to Gb3 receptors at the cell surface. The percentage of cells stained with the anti-6x-HisTag AF647 antibody is indicated on each graph. Peaks 2 and 3 exhibited the highest amount of binding to Ramos cells compared to the control. Peak 2 showed the lowest amount of binding to Namalwa cells. The fluorescence intensity is displayed in arbitrary units (AU).

To start the production process, LB-medium was inoculated with BL21(DE3) *E. coli* carrying the pET22b-StxB-scFv UCHT1 plasmid. The bacteria were grown to the desired density, and the protein expression was induced with IPTG. Expression was carried out overnight. Then, the bacteria were harvested by centrifugation. As the plasmid contained a *pelB* sequence, the protein was translocated to the periplasm. Therefore, a French press was required for bacterial cell lysis. The lysate was spun down, and the supernatant loaded onto a Ni-NTA affinity chromatography column (Figure 2A).

Figure 2B shows that the production process yielded a mixture of differently sized proteins from the affinity chromatography. The calculated size of the monomeric StxB-scFv UCHT1 lectibody is 36 kDa. In order to obtain a pure 36 kDa protein, we performed a SEC following the Ni-NTA purification. In the SEC, we observed that the highest amount of protein was located at around 180 mL, which, according to the standard curve (Supplementary Figure S7), corresponds to approximately 70 kDa (Figure 2C, peak 2) in agreement with the theoretical size of a dimeric lectibody.

The receptor specificity of the StxB In the lectibody is of utmost importance and has proven to be challenging throughout the development process. Hence, we tested the binding specificity of all peaks obtained by SEC. To this end, Ramos cells with a high abundance of Gb3, from now on called Gb3+ (Figure 2D), and Namalwa cells with a very low abundance of Gb3, from now on referred to as Gb3- (Figure 2E), were incubated with 14 nM of protein corresponding to each SEC peak. Figure 2D,E depict the fluorescence intensity of the anti-6x-HisTag AF647 antibody that recognizes the lectibody bound to Ramos and Namalwa cells. High fluorescence intensity values recorded at the flow cytometer indicate a high percentage of binding to target cells. Therefore, the binding pattern for this lectibody was expected to exhibit a high fluorescence activity for Ramos cells and a low one for Namalwa cells. Peaks 1 and 4 showed around 85% and 63% binding to Ramos cells, respectively. Both peak 2 and peak 3 displayed almost 100% binding of the lectibody to Ramos cells. On the other hand, when incubated with Namalwa cells, peaks 2, 3, and 4 revealed a similar binding, around 2%. In brief, peak 4 performed weaker binding to Ramos and Namalwa cells than peaks 2 and 3. All peaks were further tested in a bioluminescence (BLI)-based cytotoxicity assay. In addition to exhibiting reduced specificity towards target cells in flow cytometry, peaks 1 and 4 were also non-specific in terms of killing activity (Supplementary Figure S8). Peaks 2 and 3 alike showed specificity towards target cells in their binding profile. However, peak 3 induced a time and dose-dependent cytotoxicity towards Ramos cells. Upon co-incubation with Namalwa cells, the measured cytotoxicity was time- and dose-independent, suggesting unspecific T cell activation (Supplementary Figure S9). Consequently, the dimeric lectibody contained in peak 2 was used for further experiments and referred to as the StxB-scFv UCHT1 lectibody. 

### 3.3. Ability of the StxB-scFv UCHT1 Lectibody to Induce T-Cell-Mediated Cancer Cell Killing

#### 3.3.1. Burkitt’s Lymphoma Cells as a Model for Haematological Cancers

**Figure 3:**
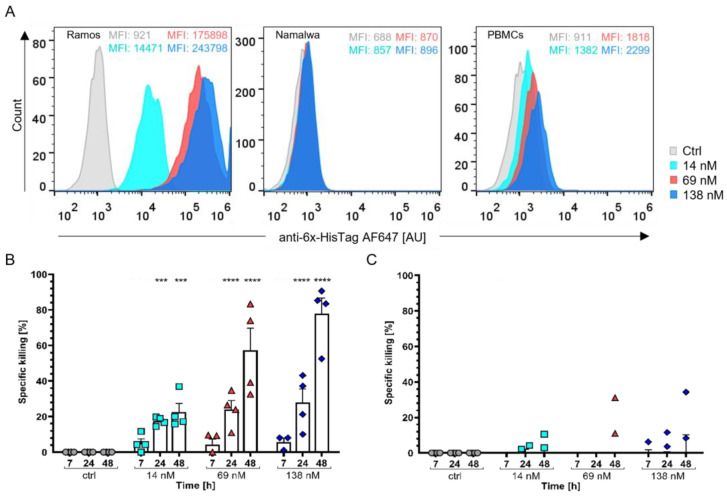
Binding of StxB-scFv UCHT1 lectibody and induced T cell cytotoxicity to Burkitt’s lymphoma-derived cells. (**A**) Representative flow cytometry histograms of Ramos cells, Namalwa cells, and PBMCs incubated with the lectibody. Cells were treated with different concentrations of lectibody and stained with a fluorescent anti-6x-HisTag AF647 antibody to detect StxB-scFv UCHT1 presence at the cell surface. A dose-dependent increase in binding to Gb3 at the surface of treated cells is visible for Gb3+ Ramos, but not for Gb3- Namalwa cells. The lectibody also bound dose-dependently to the CD3 receptors on PBMCs. Mean fluorescence (MFI) values depicted for every concentration. Cytotoxicity assays with (**B**) Ramos cells or (**C**) Namalwa cells were performed to assess StxB-scFv UCHT1-mediated T cell cytotoxicity. Cells were incubated with PBMCs in an effector to target (E:T) ratio of 5:1 for a period of 48 h. The specific killing activity (%) of PBMCs was measured. The data are shown as the mean ± SEM (*n* = 4) of four separate experiments. All experiments were performed in triplicate. (**B**) Significant killing of target cells was observed for all concentrations after 24 h of co-incubation, while at 48 h, the specific killing reached up to 78% for 138 nM StxB-scFv UCHT1. (**C**) There was no detectable specific killing of Namalwa cells in the presence of PBMCs and StxB-scFv UCHT1. For statistical analysis, the mean of each concentration was compared to the mean of the control (ctrl) at each timepoint. *n* = 4; *p*-values < 0.5 were considered significant. *** *p* ≤ 0.001; **** *p* ≤ 0.0001.

A functional landmark for the development process of the StxB-scFv UCHT1 lectibody was the evaluation of the lectibody binding efficiency and the ability to elicit lysis of cancer cells. As contemporary BiTE treatments have been proven as useful against haematological cancers and are being utilised after a relapse or the failure of standard treatments, we chose Burkitt’s lymphoma-derived cells as model cell lines [27,82]. Ramos cells (Gb3-positive, Gb3+) and Namalwa cells (Gb3-negative, Gb3-) were taken as representative cell lines for this malignancy. To validate the binding properties of the lectibody in the chosen concentrations (14, 69, 138 nM), the cells were incubated with the StxB-scFv UCHT1 lectibody and then analysed using flow cytometry. The lectibody bound to Gb3 exposed on the surface of treated cells and was detected with an anti-6x-HisTag AF647 antibody. As illustrated in Figure 3A, the lectibody bound in a dose-dependent manner to Ramos cells and PBMCs isolated from healthy donors. Only very weak binding to Namalwa cells could be detected (<2%). The binding of the lectibody to Burkitt’s lymphoma-derived cells was comparable to that observed with commercially available StxB (Supplementary Figure S10). 

To assess the efficacy of the StxB-scFv UCHT1 lectibody to redirect unstimulated, peripheral T cells to lyse Gb3+ Burkitt’s lymphoma cells, Ramos and Namalwa cells were co-cultured with PBMCs in a effector to target (E:T) ratio of 5:1 for 48 h in the presence of 14, 69, or 138 nM of the lectibody. The StxB-scFv UCHT1 lectibody induced cell death in Gb3+ Ramos cells, in a dose-dependent manner (Figure 3A). Cell death was recorded as a decrease in BLI at 7, 24, and 48 h. To monitor for spontaneous cell death, PBMCs and cancer cells were co-incubated together without the lectibody. Figure 3B,C depict data of in vitro BLI assays displaying the specific killing activity induced by the lectibody as a percentage. The StxB-scFv UCHT1 lectibody provoked T cells’ cytotoxic activity towards Gb3+ Ramos cells (Figure 3B), resulting in cancer cell elimination. At all concentrations, around 25–30% of killing activity was measured at 24 h posttreatment. After 48 h, incubation of the target and effector cells with 14 nM StxB-scFv UCHT1 lectibody derived a specific Ramos cells killing close to 25%, which substantially increased up to 60–80% with 69 nM and 138 nM of StxB-scFv UCHT1. However, no killing activity was noticed in the presence of Gb3- Namalwa cells (Figure 3C). 

The most likely dimeric StxB-scFv UCHT1 lectibody successfully demonstrated its ability to specifically target and redirect T-cell-induced lysis towards Burkitt’s lymphoma-derived cells profiled for Gb3, even at nanomolar concentrations. 

#### 3.3.2. Colon Adenocarcinoma Cells as a Model for Solid Tumour Treatment

As the number of colon cancer cases is rapidly increasing, resulting in the second-most cancer-related deaths worldwide and in cancer onset at a very young age in patients, it is of exceptional importance to find effective immunotherapeutic treatment approaches [83,84]. To this end, we tested the in vitro activity of the StxB-scFv UCHT1 lectibody against colon adenocarcinoma cell lines HT29 and LS174. In this solid tumour model, HT29 represent cells with high Gb3 abundance, while LS174 cells are characterized by a low abundance of the Gb3 antigen; they will hereafter be called Gb3+ and Gb3-, respectively [69]. 

**Figure 4:**
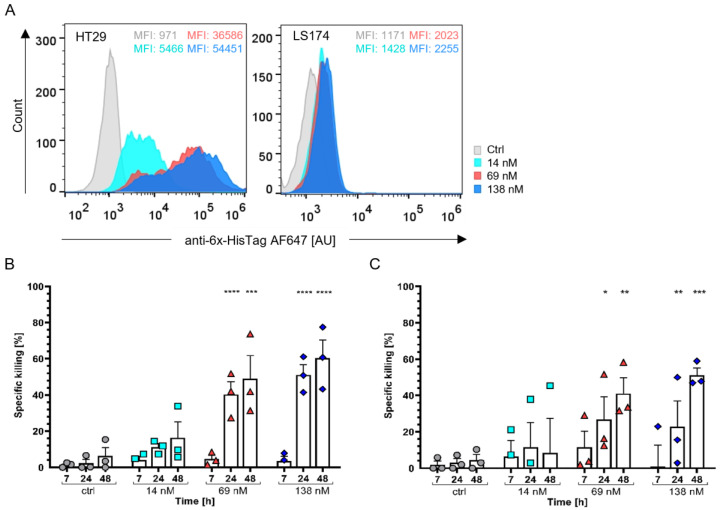
Binding and target cell lysis by StxB-scFv UCHT1 lectibody on human colon adenocarcinoma cells. (**A**) Representative flow cytometry data of lectibody-incubated HT29 and LS174 cells. Cells were incubated with different concentrations of lectibody (14, 69, 138 nM) and stained with a fluorescent anti-6x-HisTag AF647 antibody. A dose-dependent increase in binding to Gb3 at the cell surface was visible for Gb3+ cells (HT29), and for cells with low Gb3 abundance (LS174), the binding was determined to be max. 12%. MFI values depicted for each concentration. Cytotoxicity assays with (**B**) HT29 cells or (**C**) LS174 were performed to assess StxB-scFv UCHT1-mediated T cell cytotoxicity. The specific killing of T cells (%) in an effector to target (E:T) ratio of 5:1 was measured at different timepoints for a period of 48 h. The data are shown as the mean ± SEM (*n* = 3) of three separate experiments. All experiments were performed in triplicate. (**B**) After 24 h of co-incubation, significant killing of target cells could be observed for 69 and 138 nM lectibody concentrations, respectively, reaching up to 60% specific killing for 138 nM StxB-scFv UCHT1 only after 48 h. (**C**) Specific killing of the LS174 cells after 24 h at 69 and 138 nM could also be detected. *n* = 3; *p*-values < 0.5 were considered significant. * *p* ≤ 0.05; ** *p* ≤ 0.01; *** *p* ≤ 0.001; **** *p* ≤ 0.0001.

As described earlier, these cell lines were also incubated with 14, 69, and 138 nM of the lectibody and then stained with an anti-6x-HisTag AF647 antibody for flow cytometry analysis (Figure 4A). The HT29 cells exhibited high levels of StxB-scFv UCHT1 lectibody binding comparable with that of commercially available StxB (Supplementary Figure S10). We recorded up to 12% binding of the lectibody to LS174 cells, which is consistent with the binding profile of commercial StxB (Supplementary Figure S10). 

For this colon adenocarcinoma model, the StxB-scFv UCHT1 lectibody was more challenged when specifically activating T cells and redirecting tumour cell lysis. In Gb3+ HT29 cells, the lectibody (at 138 nM) induced up to 70% target cell lysis after 48 h. At this timepoint, we also registered up to 50% killing of the LS174 cells, as a consequence of their low Gb3 abundance at the surface. Notably, the effect of the lectibody towards HT29 cells was stronger (*p* < 0.0001; Figure 4B) than the impact on LS174 cells (*p* < 0.05; Figure 4C). The observed cytotoxicity induced by the lectibody on cells with a low abundance of Gb3, LS174 cells, could stem from the presence of a small percentage of cells expressing Gb3 according to Figure 4A. Rosato et al. also observed ~40% killing activity at 48 h when treating LS174 cells with their clickable pentameric Stx1B-scFv OKT3 lectibody [68]. 

### 3.4. StxB-scFv UCHT1 Lectibody Cytotoxicity Is Strictly T-Cell-Mediated

**Figure 5:**
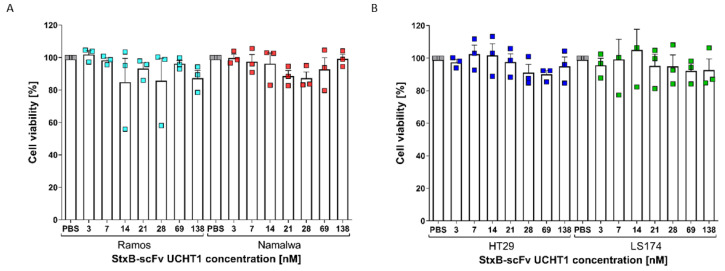
MTT assay to evaluate cell viability upon treatment of target cells with StxB-scFv UCHT1. When (**A**) Ramos and Namalwa cells or (**B**) HT29 and LS174 cells were incubated with different concentrations of StxB-scFv UCHT1 lectibody (3, 7, 14, 21, 28, 69, 138 nM) for 24 h, no toxic effects induced solely by the lectibody were detectable. Cell viability ranged between 80 and 100% after 24 h of incubation. The data are shown as the mean ± SEM (*n* = 3) of three separate experiments. *n* = 3.

To confirm that the assessed target cell death upon treatment with the lectibody could be attributed to its capability to activate and redirect peripheral T cells, we performed a cell viability assay. Burkitt’s lymphoma and colon adenocarcinoma cell lines were incubated with a broad range of concentrations of lectibody (3–138 nM) for 24 h. At the end of incubation, the 3-(4,5-Dimethylthiazol-2-yl)-2,5-diphenyltetrazoliumbromid (MTT) reagent was added, and the cells were incubated for another 4 h at 37 °C. Metabolically active cells are able to take up the yellow tetrazolium salt MTT and reduce it to purple formazan, which is then exocytosed. After solubilisation, the amount of formazan in the medium can be measured by its absorbance at 570 nm and used to estimate cell viability and proliferation [85]. In Figure 5, the data of three biological replicates are pooled. Upon treatment with the StxB-scFv UCHT1 lectibody, cell viability mostly ranged between 80 and 100% for all four tested cell lines. Hence, the data show no toxic effects exerted by the lectibody in the absence of peripheral effector T cells. These results suggest that the toxicity recorded in our BLI assays can be solely attributed to the lectibody´s ability to redirect T cells for targeted cell lysis.

## 4. Discussion

Targeting cancer cells in a highly specific manner is one of the biggest challenges in cancer therapy today. Altered glycan structures have proven to be suitable targets for immunotherapeutic approaches as they not only aid tumour progression but are also linked to the development of multidrug resistance and metastasis [35,46,86,87]. 

The lectibody generated in this study was designed after the BiTE archetype. This bispecific antibody format combines a tumour-associated antigen-binding antibody fragment with an antibody fragment specific for CD3. The most prominent example of this class of therapeutics is blinatumomab, a CD19 × CD3 bispecific antibody, which is used to effectively clear B-cell malignancies [88,89,90]. Here, we suggest the use of the lectin StxB to target with high specificity the glycosphingolipid Gb3, which is highly abundant in a multitude of cancer types [91,92,93]. In this study, we aimed to couple a monomer of StxB to a scFv αCD3 to effectively redirect and activate peripheral T cells.

One of the most crucial steps in the development of this lectibody was to ensure the stability of the StxB monomer by preventing steric hindrance or issues related to the correct folding of the two domains.

An essential factor in the design of a fusion protein such as this is the stability of the individual protein components in the resulting chimera. It is known that the conformation of monomeric StxB would be highly unstable because of the large entropy penalty of the monomer that cannot be compensated by the enthalpy gain. The interface of intermonomeric contacts is highly hydrophobic (65% of the interacting surfaces contain apolar groups) [80] and tends to be hidden from the contacts with water in the course of oligomerization. Nevertheless, other studies demonstrated that the fusion of monomeric StxB to a partner would result in a stable and functional protein [71]. In the current study, we used computational modelling to rationally design a fusion protein where StxB and scFv monomer modules were connected via a linker of appropriate length and flexibility. This allows the hydrophobic patches on the StxB surface to be hidden from water via protein–protein interactions that prevent the pentamer StxB formation, at the same time, keeping the Gb3-binding site intact, which is important to maintain StxB´s ability to bind Gb3 [94,95]. The desired monomeric StxB-scFv fusion protein was constructed in silico, and both folding and protein–protein interactions in the lectibody were proven to be stable in the course of MD trajectory. Furthermore, only slight modifications in the predicted geometry of the StxB-scFv fusion protein were shown to drive the monomers to a stable dimer formation.

The poor performance of the monomeric lectibody to induce specific killing compared with the dimeric version (Supplementary Figure S9) could be attributed to unstabilised hydrophobic regions in the StxB monomer once the αCD3 is bound to the T cells. 

The unstabilised hydrophobic regions of StxB come into play when the scFv fragment is bound to the CD3 receptor on the T cell surface. The antibody fragment can no longer flop downwards to cover and stabilise the hydrophobic regions of StxB, leaving them exposed to unspecifically stick to the surface of target cells. Hence, the observed killing is Gb3 independent and does not relate to the time of incubation or the dose applied as can be seen in Supplementary Figure S9. This problem is circumvented by dimerization, as one scFv can always cover the hydrophobic regions of the StxB and stabilise them. In this way, one αCD3 can still bind to the T cell. As this effect would only be relevant when both target and effector cells are present, it would not be visible in the flow cytometry analysis because only one type of cell is incubated with the lectibody (Supplementary Figure S9 and Figure 2D,E).

The StxB-scFv UCHT1 lectibody was expressed in BL21(DE3) *E. coli* using the pET-22b plasmid. *E. coli* is a cell factory that has evolved into the most widely used expression system [96]. The natural pathway for StxB after expression is the transport to the periplasm where it assembles as a pentamer [97]. The oxidizing environment in the periplasm is also needed for the correct folding of scFv antibodies in *E. coli* [98,99,100]. Without the oxidizing environment that the periplasm provides, neither the antibody fragment nor the StxB fragment of the lectibody would be able to form the necessary disulphide bonds they need to fold correctly [101,102,103,104]. Therefore, the plasmid used in this study included a pelB sequence translocating the lectibody to the periplasm after expression (Supplementary Figure S2). 

The protein yield was optimal when using 0.5 mM IPTG to induce the expression at an OD of 0.6 and carrying out the expression overnight at 20 °C. Firstly, the protein was purified using a 6x HisTag at the N-terminal end of the lectibody. Ni-NTA affinity purification led to a mixture of different-sized proteins as seen in Figure 2A. Therefore, we performed a SEC following the first purification step (Figure 2A). A mixture of proteins with variable sizes after affinity purification (Figure 2B) could be traced back to an array of different reasons. The most likely reason in our case is cross-contamination of the StxB-scFv UCHT1 with misfolded proteins from the cytoplasmic fraction during cell lysis [105]. Therefore, the lysis step could be improved by using a different lysis buffer composition or freeze–thaw cycles [106,107]. After SEC, we tested the purified proteins for their binding specificity to Gb3 on the surface of Gb3-positive and -negative cancer cells and for their ability to induce specific T cell cytotoxicity. The obtained proteins exhibited varying specificities towards Gb3. The protein corresponding to the calculated molecular weight of a dimeric StxB-scFv UCHT1 lectibody (70 kDa) proved to be the most specific one (Figure 2C). 

As several cancer types were reported to overexpress Gb3 and have been linked to multidrug resistance, invasiveness, and metastasis [46,51,52], a haematological (Burkitt’s lymphoma) and a solid cancer (colon adenocarcinoma) model were chosen for the present study. The lectibody displayed binding to Burkitt’s lymphoma cells with a high abundance of Gb3, namely Ramos cells, in a dose-dependent manner but not to Namalwa cells, characterised by a low abundance of Gb3 (Figure 3A). Binding of StxB-scFv UCHT1 to PBMCs could also be observed in a dose-dependent manner as highlighted in Figure 3A; however, binding was lower than compared to commercial αCD3 antibodies (Supplementary Figure S11). The StxB-scFv UCHT1 lectibody relies on a dimeric StxB to target Gb3. While pentameric StxB has strong avidity for membrane-presented Gb3 (Kd~4.2 nM) [108], the affinity for the trisaccharide in solution is much weaker (Kd~0.5 mM) [109]. Such low affinity is expected for a monomeric and a dimeric StxB-scFv UCHT1 lectibody. As seen in SPR measurements, the affinity of the lectibody was not great enough to be measured via SPR and the lectibody dissociated from the chip (Supplementary Figure S12). The lectibody was able to redirect T cells to lyse tumour cells when Gb3+ Ramos cells were co-incubated with PBMCs. With Namalwa cells, which exhibit low amounts of Gb3 at the surface, there was no apparent tumour cell lysis induced by treatment with the lectibody (Figure 3C). When comparing the StxB-scFv UCHT1 lectibody to the pentameric version developed by Rosato et al., the lectibody presented in this study revealed an efficacy that was about 15% lower with the highest percentage of killing being ~80% (Figure 3B) [68]. This can be explained by the fact that this lectibody contains a dimeric StxB, whereas the clickable version from Rosato et al. contains a pentameric StxB. The lectibody presented here possessed two UCHT1 αCD3 per lectibody, while the pentameric version contained three αCD3 scFvs. Moreover, the pentameric version utilized an OKT3 scFv that has been shown to have a higher affinity to CD3 than the UCHT1 scFv [110,111]. The combination of the higher affinity and the higher number of αCD3 antibody fragments could potentially lead to an overactivation of CD4+ and CD8+ T cells, which might result in T cell exhaustion and, ultimately, apoptosis of the coupled T cells [112]. It is also known that UCHT1 in comparison with OKT3 induces activation of T cells via the CD3 receptor more effectively and, in turn, improves the capabilities of the T cell to induce target cell lysis. In a similar study, Dopfer et al. demonstrated that UCHT1 induced T cell-mediated target cell lysis at much lower concentrations than OKT3 [113].

Moreover, the design of the StxB-scFv UCHT1 lectibody as a fusion protein was much simpler in comparison to the StxB-scFv OKT3 lectibody, which was expressed as separate proteins and subsequently assembled into a lectibody format by click chemistry. In particular, the production process of the StxB-scFv UCHT1 lectibody was simplified and reduced to fewer steps. Nevertheless, the design of this format as a fusion protein and its expression in a bacterial host also carries its drawbacks. For one, there is no possibility to influence pentamer formation in the periplasm. As visible from the docking model in Supplementary Figure S6, the monomeric StxB tends to interact with the UCHT1, hindering pentamer formation. The use of a short linker constituted by only four amino acids does not allow the further shortening of the linkage between StxB and UCHT1 to prevent the interaction of the StxB monomer with the UCHT1 in solution and favour pentamer formation. Additionally, the ideal exchange of domains in the lectibody platform, to target another TACA for example, would be more complex in this strategy. The exchange of the sequence in the plasmid would require arguably more effort than to click another lectin to the existing UCHT1. When designing recombinant fusion proteins such as the StxB-scFv UCHT1 lectibody, it is essential to consider the different expression conditions required for the individual components. As mentioned earlier, antibodies need an oxidizing environment during expression in order to undergo proper folding and formation of disulphide bonds [101,102,103,104]. Combining a lectin that needs the reducing conditions of the cytoplasm with a scFv requiring oxidizing conditions of the periplasm might therefore prove impossible.

When tested in combination with a solid tumour model, the StxB-scFv UCHT1 lectibody elicited less tumour cell lysis of Gb3+ HT29 cells compared to Gb3+ Ramos cells. It is widely known that solid tumours present major hurdles for immunotherapeutic approaches [114,115,116]. The tumour microenvironment (TME) is generally immunosuppressive, as many tumours release immunosuppressive cytokines and chemokines [114]. The combination of BiTE administration with chemotherapeutic administration has been shown to be helpful to overcome this challenge [114,117]. Solid tumours also pose a challenge to drug delivery and penetration of the tumour. In pursuit of a possibility to overcome this problem, Scott and colleagues armed oncolytic viruses with BiTEs, thereby increasing the therapeutic window and joining it with direct oncolysis [115]. As the lectibody design is based on the design of BiTEs, it is likely that approaches of the same kind could greatly improve the efficacy of lectibodies as well. In our study, binding of the lectibody to Gb3+ colon adenocarcinoma cells in solution resulted in being very efficient and comparable to the binding of StxB (Figure 4A and Figure S10) [49,118]. However, the Gb3- LS174 cells showed a basal expression of Gb3 leading to a binding of the lectibody of up to 12% (Figure 4A). When exposed to the lectibody for longer periods of time, this relatively basal amount of Gb3 was enough to induce LS174 cell lysis [119,120,121,122]. We could prove that the killing of LS174 cells is due to the activation of peripheral T cells as the lectibody does not affect cell viability (Figure 5B). However, the effect was less significant (*p* < 0.05) than the one on cells with a high Gb3 abundance, namely HT29 cells (*p* < 0.0001). Those results are in accordance with the effects reported by Rosato et al. [68]. This raises the question about off-target effects in an in vivo model. Gb3 is present in low abundance on intestinal endothelial cells, kidney cells, and in the brain [123,124,125]. As the lectibody induced target cell lysis in cells with low Gb3 abundance, it is possible that off-target effects in tissues with low Gb3 abundance might occur in vivo. As with any new immunotherapeutic agent, off-target toxicity would have to be monitored and the treatment dose adjusted to a rate where the off-target toxicity is at a minimum. For solid tumours, it is also possible to circumvent this issue by injecting the lectibody directly to the tumour site, which would reduce off-target effects to a minimum. This habit of recognizing cancer cells with low Gb3 abundance can also be an advantage for the treatment with the lectibody, as it can reduce the chances that the cancer cells can use antigen escape to avoid clearance by T cells. Furthermore, the lectibody has a size of 70 kDa, which is similar to the size of comparable molecules such as BiTEs, diabodies, or tandem scFv, which range between 50 and 60 kDa in size [126,127]. These molecules are typically cleared from circulation within hours, making it necessary for multiple injections or the use of infusions for the treatment administration [128,129,130]. While this makes it more challenging to get the molecule to the site of action, it can also allow a more precise dosing. The clearance rate for the lectibody would have to be determined in in vivo studies. 

It has been observed that, among the cell lines used in this study, HT29 cells exhibit the highest amount of Gb3 on the cell surface, closely followed by Ramos cells. Namalwa and LS174 only showed small amounts of Gb3. The Gb3 isoforms indicated in HT29 were mainly hydroxylated isoforms (C16:0-OH, C24:0-OH), which in the past have been connected to augmented binding of StxB [131]. On the other hand, the most abundant forms in Ramos cells were non-saturated C24:1, closely followed by saturated C16:0 and C24:0 [69,131]. It was previously demonstrated that the presence of Gb3 with unsaturated fatty acyl chains increases the binding of StxB [132]. This correlates with the findings reported in the present study, where binding and cytotoxicity of the lectibody were greatest in the presence of Ramos cells. In the study performed by Meléndez et al., a higher abundance of Gb3 on the surface of tumour cells was correlated to a higher cytotoxic effect of the studied CAR T cells [69]. Gb3 levels are known to oscillate depending on the cell cycle and differentiation of the cells; therefore, the cytotoxicity exhibited from LS174 cells [133]. 

In conclusion, the StxB-scFv UCHT1 lectibody successfully developed in this study showed specific binding to Gb3+ Ramos lymphoma cells and HT29 colon cancer cells. Notably, it did not bind to Gb3- Namalwa and LS174 cells or did so only in low levels. During co-incubation of target cells with PBMCs, the lectibody specifically induced T-cell-dependent target cell lysis in Burkitt’s lymphoma cells over a wide range of concentrations (14, 69, 138 nM), whereas with colon adenocarcinoma cells, the lectibody was able to induce T-cell-mediated target cell lysis in Gb3+ HT29 cells. 

In this research, we further demonstrated the ability of lectins to be applied in immunotherapy, effectively and specifically targeting different types of cancer cells with a high Gb3 abundance. Given the limited treatments available for cancer, lectibodies and other lectin-based tools open up new therapeutic possibilities.

## Data Availability

Data are contained within the article or Supplementary Material. The data presented in this study are available upon reasonable request.

## Supplementary Materials

The following supporting information can be downloaded at: https://www.mdpi.com/article/10.3390/cells12141896/s1, Figure S1: StxB-scFv UCHT1 sequence; Figure S2: pET22b plasmid map; Figure S3: Amino acid sequence of the StxB-scFv UCHT1 lectibody; Figure S4: Protein–protein docking poses; Figure S5: Root-mean-square deviation analysis; Figure S6: Equilibrated StxB-scFv UCHT1 in silico structure; Figure S7: Standard curve SEC to determine protein size; Figure S8: Killing assay peaks 1 and 4; Figure S9: Binding of StxB-scFv UCHT1 lectibody and induced cytotoxicity by peak 3; Figure S10: StxB binding by target cell lines; Figure S11: PBMCs binding of αCD3; Figure S12: Representative SPR data of peaks 2 and 3; Table S1: Per-residue contacts between StxB and scFv UCHT1.

## References

[1] Sung H., Ferlay J., Siegel R.L., Laversanne M., Soerjomataram I., Jemal A., Bray F. (2021). Global Cancer Statistics 2020: GLOBOCAN Estimates of Incidence and Mortality Worldwide for 36 Cancers in 185 Countries. CA Cancer J. Clin..

[2] Wildgaard K., Ravn J., Nikolajsen L., Jakobsen E., Jensen T.S., Kehlet H. (2011). Consequences of persistent pain after lung cancer surgery: A nationwide questionnaire study. Acta Anaesthesiol. Scand..

[3] Tohme S., Simmons R.L., Tsung A. (2017). Surgery for Cancer: A Trigger for Metastases. Cancer Res..

[4] Carter J., Stabile C., Gunn A., Sonoda Y. (2013). The Physical Consequences of Gynecologic Cancer Surgery and Their Impact on Sexual, Emotional, and Quality of Life Issues. J. Sex. Med..

[5] Lindley C.M., Hirsch J.D., O’Neill C.V., Transau M.C., Gilbert C.S., Osterhaus J.T. (1992). Quality of life consequences of chemotherapy-induced emesis. Qual. Life Res..

[6] Baskar R., Lee K.A., Yeo R., Yeoh K.W. (2012). Cancer and Radiation Therapy: Current Advances and Future Directions. Int. J. Med. Sci..

[7] Waldman A.D., Fritz J.M., Lenardo M.J. (2020). A guide to cancer immunotherapy: From T cell basic science to clinical practice. Nat. Rev. Immunol..

[8] Mellman I., Coukos G., Dranoff G. (2011). Cancer immunotherapy comes of age. Nature.

[9] Till S.J., Francis J.N., Nouri-Aria K., Durham S.R. (2004). Mechanisms of immunotherapy. J. Allergy Clin. Immunol..

[10] Köhler G., Milstein C. (1975). Continuous cultures of fused cells secreting antibody of predefined specificity. Nature.

[11] Kontermann R.E., Brinkmann U. (2015). Bispecific antibodies. Drug Discov. Today.

[12] Bird R.E., Walker B.W. (1991). Single chain antibody variable regions. Trends Biotechnol..

[13] Ahamadi-Fesharaki R., Fateh A., Vaziri F., Solgi G., Siadat S.D., Mahboudi F., Rahimi-Jamnani F. (2019). Single-Chain Variable Fragment-Based Bispecific Antibodies: Hitting Two Targets with One Sophisticated Arrow. Mol. Ther.-Oncolytics.

[14] Kelly M.P., Lee F.T., Tahtis K., Power B.E., Smyth F.E., Brechbiel M.W., Hudson P.J., Scott A.M. (2008). Tumor Targeting by a Multivalent Single-Chain Fv (scFv) Anti-Lewis Y Antibody Construct. Cancer Biother. Radiopharm..

[15] Chowdhury P.S., Viner J.L., Beers R., Pastan I. (1998). Isolation of a high-affinity stable single-chain Fv specific for mesothelin from DNA-immunized mice by phage display and construction of a recombinant immunotoxin with anti-tumor activity. Proc. Natl. Acad. Sci. USA.

[16] Huehls A.M., Coupet T.A., Sentman C.L. (2015). Bispecific T-cell engagers for cancer immunotherapy. Immunol. Cell Biol..

[17] Baeuerle P.A., Reinhardt C. (2009). Bispecific T-Cell Engaging Antibodies for Cancer Therapy. Cancer Res..

[18] Wolf E., Hofmeister R., Kufer P., Schlereth B., Baeuerle P.A. (2005). BiTEs: Bispecific antibody constructs with unique anti-tumor activity. Drug Discov. Today.

[19] Zhou S., Liu M., Ren F., Meng X., Yu J. (2021). The landscape of bispecific T cell engager in cancer treatment. Biomark. Res..

[20] Baeuerle P.A., Kufer P., Bargou R. (2009). BiTE: Teaching antibodies to engage T-cells for cancer therapy. Curr. Opin. Mol. Ther..

[21] Slaney C.Y., Wang P., Darcy P.K., Kershaw M.H. (2018). CARs versus BiTEs: A Comparison between T Cell–Redirection Strategies for Cancer Treatment. Cancer Discov..

[22] Trapani J.A., Smyth M.J. (2002). Functional significance of the perforin/granzyme cell death pathway. Nat. Rev. Immunol..

[23] Haas C., Krinner E., Brischwein K., Hoffmann P., Lutterbüse R., Schlereth B., Kufer B., Baeurele P.A. (2009). Mode of cytotoxic action of T cell-engaging BiTE antibody MT110. Immunobiology.

[24] Osińska I., Popko K., Demkow U. (2014). Perforin: An important player in immune response. Cent. Eur. J. Immunol..

[25] Zhan Y., Carrington E.M., Zhang Y., Heinzel S., Lew A.M. (2017). Life and Death of Activated T Cells: How Are They Different from Naïve T Cells?. Front. Immunol..

[26] Zhang J.M., An J. (2007). Cytokines, Inflammation, and Pain. Int. Anesthesiol. Clin..

[27] Przepiorka D., Ko C.W., Deisseroth A., Yancey C.L., Candau-Chacon R., Chiu H.-J., Gehrke B.J., Gomez-Broughton C., Kirshner S. (2015). FDA Approval: Blinatumomab. Clin. Cancer Res..

[28] Nagorsen D., Kufer P., Baeuerle P.A., Bargou R. (2012). Blinatumomab: A historical perspective. Pharmacol. Ther..

[29] Nair-Gupta P., Diem M., Reeves D., Wang W., Schulingkamp R., Sproesser K., Mattson B., Heidrich B., Mendonca M., Joseph J. (2020). A novel C2 domain binding CD33xCD3 bispecific antibody with potent T-cell redirection activity against acute myeloid leukemia. Blood Adv..

[30] Subklewe M., Stein A., Walter R.B., Bhatia R., Wei A.H., Ritchie D., Bücklein V., Vachhani P., Dai T., Hindoyan A. (2019). Preliminary Results from a Phase 1 First-in-Human Study of AMG 673, a Novel Half-Life Extended (HLE) Anti-CD33/CD3 BiTE^®^ (Bispecific T-Cell Engager) in Patients with Relapsed/Refractory (R/R) Acute Myeloid Leukemia (AML). Blood.

[31] Hummel H.D., Kufer P., Grüllich C., Seggewiss-Bernhardt R., Deschler-Baier B., Chatterjee M., Goebeler M.-E., Miller K., de Santis M., Loidl W. (2021). Pasotuxizumab, a BiTE ^®^ immune therapy for castration-resistant prostate cancer: Phase I, dose-escalation study findings. Immunotherapy.

[32] Sternjak A., Lee F., Thomas O., Balazs M., Wahl J., Lorenczewski G., Ullrich I., Muenz M., Rattel B., Bailis J.M. (2021). Preclinical Assessment of AMG 596, a Bispecific T-cell Engager (BiTE) Immunotherapy Targeting the Tumor-specific Antigen EGFRvIII. Mol. Cancer Ther..

[33] Hanahan D., Weinberg R.A. (2000). The Hallmarks of Cancer. Cell.

[34] Hakomori S.I. (1991). Possible functions of tumor-associated carbohydrate antigens. Curr. Opin. Immunol..

[35] Dube D.H., Bertozzi C.R. (2005). Glycans in cancer and inflammation—Potential for therapeutics and diagnostics. Nat. Rev. Drug Discov..

[36] Zhuo D., Li X., Guan F. (2018). Biological Roles of Aberrantly Expressed Glycosphingolipids and Related Enzymes in Human Cancer Development and Progression. Front. Physiol..

[37] Hakomori S itiroh Kannagi R. (1983). Glycosphingolipids as Tumor-Associated and Differentiation Markers56. JNCI J. Natl. Cancer Inst..

[38] Pellizzari A., Pang H., Lingwood C.A. (1992). Binding of verocytotoxin 1 to its receptor is influenced by differences in receptor fatty acid content. Biochemistry.

[39] Merrill A.H. (2011). Sphingolipid and glycosphingolipid metabolic pathways in the era of sphingolipidomics. Chem. Rev..

[40] Siukstaite L., Imberty A., Römer W. (2021). Structural Diversities of Lectins Binding to the Glycosphingolipid Gb3. Front. Mol. Biosci..

[41] Okuda T., Tokuda N., Numata S.I., Ito M., Ohta M., Kawamura K., Wiels J., Urano T., Tajima O. (2006). Targeted Disruption of Gb3/CD77 Synthase Gene Resulted in the Complete Deletion of Globo-series Glycosphingolipids and Loss of Sensitivity to Verotoxins*. J. Biol. Chem..

[42] Okuda T., Nakayama K.I. (2008). Identification and characterization of the human Gb3/CD77 synthase gene promoter. Glycobiology.

[43] Kannagi R., Fukuda M.N., Hakomori S. (1982). A new glycolipid antigen isolated from human erythrocyte membranes reacting with antibodies directed to globo-N-tetraosylceramide (globoside). J. Biol. Chem..

[44] Hardie D.L., Johnson G.D., Khan M., MacLennan I.C. (1993). Quantitative analysis of molecules which distinguish functional compartments within germinal centers. Eur. J. Immunol..

[45] Mangeney M., Lingwood C.A., Taga S., Caillou B., Tursz T., Wiels J. (1993). Apoptosis Induced in Burkitt’s Lymphoma Cells via Gb3/CD77, a Glycolipid Antigen1. Cancer Res..

[46] Kovbasnjuk O., Mourtazina R., Baibakov B., Wang T., Elowsky C., Choti M.A., Kane A., Donowitz M. (2005). The glycosphingolipid globotriaosylceramide in the metastatic transformation of colon cancer. Proc. Natl. Acad. Sci. USA.

[47] Geyer P.E., Maak M., Nitsche U., Perl M., Novotny A., Slotta-Huspenina J., Dransart E., Holtorf A., Johannes L., Janssen K.-P. (2016). Gastric Adenocarcinomas Express the Glycosphingolipid Gb3/CD77: Targeting of Gastric Cancer Cells with Shiga Toxin B-Subunit. Mol. Cancer Ther..

[48] Johansson D., Kosovac E., Moharer J., Ljuslinder I., Brännström T., Johansson A., Behnam-Motlagh P. (2009). Expression of verotoxin-1 receptor Gb3 in breast cancer tissue and verotoxin-1 signal transduction to apoptosis. BMC Cancer.

[49] Maak M., Nitsche U., Keller L., Wolf P., Sarr M., Thiebaud M., Rosenberg R., Langer R., Kleef J., Friess H. (2011). Tumor-Specific Targeting of Pancreatic Cancer with Shiga Toxin B-Subunit. Mol. Cancer Ther..

[50] Distler U., Souady J., Hülsewig M., Drmić-Hofman I., Haier J., Friedrich A.W., Karch H., Senninger N., Dreisewerd K., Berenkamp S. (2009). Shiga Toxin Receptor Gb3Cer/CD77: Tumor-Association and Promising Therapeutic Target in Pancreas and Colon Cancer. PLoS ONE.

[51] Behnam-Motlagh P., Tyler A., Grankvist K., Johansson A. (2010). Verotoxin-1 Treatment or Manipulation of its Receptor Globotriaosylceramide (Gb3) for Reversal of Multidrug Resistance to Cancer Chemotherapy. Toxins.

[52] Arab S., Russel E., Chapman W.B., Rosen B., Lingwood C.A. (1997). Expression of the verotoxin receptor glycolipid, globotriaosylceramide, in ovarian hyperplasias. Oncol. Res..

[53] Nativi C., Papi F., Roelens S. (2019). Tn antigen analogues: The synthetic way to “upgrade” an attracting tumour associated carbohydrate antigen (TACA). Chem. Commun..

[54] Guo Z., Wang Q. (2009). Recent development in carbohydrate-based cancer vaccines. Curr. Opin. Chem. Biol..

[55] Beatty G.L., Gladney W.L. (2015). Immune escape mechanisms as a guide for cancer immunotherapy. Clin. Cancer Res..

[56] Bates J.P., Derakhshandeh R., Jones L., Webb T.J. (2018). Mechanisms of immune evasion in breast cancer. BMC Cancer.

[57] Trofa A.F., Ueno-Olsen H., Oiwa R., Yoshikawa M. (1999). Kiyoshi Shiga: Discoverer of the dysentery bacillus. Clin. Infect. Dis..

[58] Gyles C.L. (2007). Shiga toxin-producing Escherichia coli: An overview1. J. Anim. Sci..

[59] Hasan I., Sugawara S., Fujii Y., Koide Y., Terada D., Iimura N., Fujiwara T., Takahashi K.G., Kojima N., Rajia S. (2015). MytiLec, a Mussel R-Type Lectin, Interacts with Surface Glycan Gb3 on Burkitt’s Lymphoma Cells to Trigger Apoptosis through Multiple Pathways. Mar. Drugs.

[60] Terada D., Voet A.R.D., Noguchi H., Kamata K., Ohki M., Addy C., Fujii Y., Yamamoto D., Ozeki Y., Tame J.R.H. (2017). Computational design of a symmetrical β-trefoil lectin with cancer cell binding activity. Sci. Rep..

[61] Johannes L., Römer W. (2010). Shiga toxins—From cell biology to biomedical applications. Nat. Rev. Microbiol..

[62] Morabito S. (2014). Pathogenic Escherichia Coli: Molecular and Cellular Microbiology.

[63] Römer W., Berland L., Chambon V., Gaus K., Windschiegl B., Tenza D., Aly M.R.E., Fraiser V., Florent J.-C., Perrais D. (2007). Shiga toxin induces tubular membrane invaginations for its uptake into cells. Nature.

[64] Römer W., Pontani L.L., Sorre B., Rentero C., Berland L., Chambon V., Lamaze C., Bassereau P., Sykes C., Gaus K. (2010). Actin Dynamics Drive Membrane Reorganization and Scission in Clathrin-Independent Endocytosis. Cell.

[65] Kociurzynski R., Makshakova O.N., Knecht V., Römer W. (2021). Multiscale Molecular Dynamics Studies Reveal Different Modes of Receptor Clustering by Gb3-Binding Lectins. J. Chem. Theory Comput..

[66] Donohue-Rolfe A., Jacewicz M., Keusch G.T. (1989). Isolation and characterization of functional Shiga toxin subunits and renatured holotoxin. Mol. Microbiol..

[67] Batisse C., Dransart E., Ait Sarkouh R., Brulle L., Bai S.-K., Godefroy S., Johannes L., Schmidt F. (2015). A new delivery system for auristatin in STxB-drug conjugate therapy. Eur. J. Med. Chem..

[68] Rosato F., Pasupuleti R., Tomisch J., Meléndez A.V., Kolanovic D., Makshakova O.N., Wiltschi B., Römer W. (2022). A bispecific, crosslinking lectibody activates cytotoxic T cells and induces cancer cell death. J. Transl. Med..

[69] Meléndez A.V., Velasco Cárdenas R.M.H., Lagies S., Strietz J., Siukstaite L., Thomas O.S., Tomisch J., Weber W., Kammerer B., Römer W. (2022). Novel lectin-based chimeric antigen receptors target Gb3-positive tumour cells. Cell Mol. Life Sci..

[70] Danielewicz N., Rosato F., Tomisch J., Gräber J., Wiltschi B., Striedner G., Römer W., Mairhofer J. (2023). Clickable Shiga Toxin B Subunit for Drug Delivery in Cancer Therapy. ACS Omega.

[71] Mohseni Z., Sedighian H., Halabian R., Amani J., Behzadi E., Imani Fooladi A.A. (2021). Potent in vitro antitumor activity of B-subunit of Shiga toxin conjugated to the diphtheria toxin against breast cancer. Eur. J. Pharmacol..

[72] Šali A., Blundell T.L. (1993). Comparative Protein Modelling by Satisfaction of Spatial Restraints. J. Mol. Biol..

[73] Desta I.T., Porter K.A., Xia B., Kozakov D., Vajda S. (2020). Performance and Its Limits in Rigid Body Protein-Protein Docking. Structure.

[74] Kozakov D., Hall D.R., Xia B., Porter K.A., Padhorny D., Yueh C., Beglov D., Vajda S. (2017). The ClusPro web server for protein–protein docking. Nat. Protoc..

[75] Case D.A., Ben-Shalom I.Y., Brozell S.R., Cerutti D.S., Cheatham T.E., Cruzeiro V.W.D., Darden T.A., Duke R.E., Ghoreishi D., Gilson M.K. (2018). AMBER 18.

[76] Ryckaert J.P., Ciccotti G., Berendsen H.J.C. (1977). Numerical integration of the cartesian equations of motion of a system with constraints: Molecular dynamics of n-alkanes. J. Comput. Phys..

[77] Essmann U., Perera L., Berkowitz M.L., Darden T., Lee H., Pedersen L.G. (1995). A smooth particle mesh Ewald method. J. Chem. Phys..

[78] Brischwein K., Parr L., Pflanz S., Volkland J., Lumsden J., Klinger M., Locher M., Hammond S.A., Kiener P., Kufer P. (2007). Strictly Target Cell-dependent Activation of T Cells by Bispecific Single-chain Antibody Constructs of the BiTE Class. J. Immunother..

[79] Minguet S., Swamy M., Alarcón B., Luescher I.F., Schamel W.W.A. (2007). Full Activation of the T Cell Receptor Requires Both Clustering and Conformational Changes at CD3. Immunity.

[80] Pina D.G., Gómez J., Villar E., Johannes L., Shnyrov V.L. (2003). Thermodynamic Analysis of the Structural Stability of the Shiga Toxin B-Subunit. Biochemistry.

[81] Ling H., Boodhoo A., Hazes B., Cummings M.D., Armstrong G.D., Brunton J.L., Read R.J. (1998). Structure of the Shiga-like Toxin I B-Pentamer Complexed with an Analogue of Its Receptor Gb_3_. Biochemistry.

[82] Batlevi C.L., Matsuki E., Brentjens R.J., Younes A. (2016). Novel immunotherapies in lymphoid malignancies. Nat. Rev. Clin. Oncol..

[83] Sinicrope F.A. (2022). Increasing Incidence of Early-Onset Colorectal Cancer. N. Engl. J. Med..

[84] Siegel R.L., Jakubowski C.D., Fedewa S.A., Davis A., Azad N.S. (2020). Colorectal Cancer in the Young: Epidemiology, Prevention, Management. Am. Soc. Clin. Oncol. Educ. Book.

[85] Liu Y., Peterson D.A., Kimura H., Schubert D. (2002). Mechanism of Cellular 3-(4,5-Dimethylthiazol-2-yl)-2,5-Diphenyltetrazolium Bromide (MTT) Reduction. J. Neurochem..

[86] Liu Y.Y., Gupta V., Patwardhan G.A., Bhinge K., Zhao Y., Mehendale H., Cabot M.C., Li Y.-T., Jazwinski S.M. (2010). Glycosylceramide Synthase Upregulates MDR1 Expression in the Regulation of Cancer Drug Resistance through cSrc and β-Catenin Signaling. Mol. Cancer.

[87] Padler-Karavani V. (2014). Aiming at the sweet side of cancer: Aberrant glycosylation as possible target for personalized-medicine. Cancer Lett..

[88] Feucht J., Kayser S., Gorodezki D., Hamieh M., Döring M., Blaescke F., Schlegel P., Bösmüller H., Quintanilla-Fend L., Ebinger M. (2016). T-cell responses against CD19^+^ pediatric acute lymphoblastic leukemia mediated by bispecific T-cell engager (BiTE) are regulated contrarily by PD-L1 and CD80/CD86 on leukemic blasts. Oncotarget.

[89] Zugmaier G., Klinger M., Schmidt M., Subklewe M. (2015). Clinical overview of anti-CD19 BiTE^®^ and ex vivo data from anti-CD33 BiTE^®^ as examples for retargeting T cells in hematologic malignancies. Mol. Immunol..

[90] Löffler A., Gruen M., Wuchter C., Schriever F., Kufer P., Dreier T., Baeuerle P.A., Bommert K., Karaawajew L., Dörken B. (2003). Efficient elimination of chronic lymphocytic leukaemia B cells by autologous T cells with a bispecific anti-CD19/anti-CD3 single-chain antibody construct. Leukemia.

[91] Pochechueva T., Jacob F., Fedier A., Heinzelmann-Schwarz V. (2012). Tumor-Associated Glycans and Their Role in Gynecological Cancers: Accelerating Translational Research by Novel High-Throughput Approaches. Metabolites.

[92] Hakomori S.I., Wu A.M. (2001). Tumor-Associated Carbohydrate Antigens Defining Tumor Malignancy: Basis for Development of Anti-Cancer Vaccines. The Molecular Immunology of Complex Carbohydrates—2.

[93] Engedal N., Skotland T., Torgersen M.L., Sandvig K. (2011). Shiga toxin and its use in targeted cancer therapy and imaging: Shiga toxin in cancer therapy and imaging. Microb. Biotechnol..

[94] Wang Q., Chen Y., Park J., Liu X., Hu Y., Wang T., McFArlad K., Betenbaugh M.J. (2019). Design and Production of Bispecific Antibodies. Antibodies.

[95] Brinkmann U., Kontermann R.E. (2017). The making of bispecific antibodies. mAbs.

[96] Rosano G.L., Ceccarelli E.A. (2014). Recombinant protein expression in Escherichia coli: Advances and challenges. Front. Microbiol..

[97] Kim S.H., Ryu S.H., Lee S.H., Lee Y.-H., Lee S.-R., Huh J.-W., Kim S.-U., Kim E., Kim S., Jon S. (2011). Instability of toxin A subunit of AB5 toxins in the bacterial periplasm caused by deficiency of their cognate B subunits. Biochim. Biophys. Acta BBA-Biomembr..

[98] Le Gall F., Bové J.M., Garnier M. (1998). Engineering of a Single-Chain Variable-Fragment (scFv) Antibody Specific for the Stolbur Phytoplasma (Mollicute) and Its Expression in *Escherichia coli* and Tobacco Plants. Appl. Env. Microbiol..

[99] Dewi K.S. (2016). Construction and Periplasmic Expression of the Anti-EGFRvIII ScFv Antibody Gene in Escherichia coli. Sci. Pharm..

[100] Miller K.D., Weaver-Feldhaus J., Gray S.A., Siegel R.W., Feldhaus M.J. (2005). Production, purification, and characterization of human scFv antibodies expressed in Saccharomyces cerevisiae, Pichia pastoris, and Escherichia coli. Protein Expr. Purif..

[101] Denoncin K., Collet J.F. (2013). Disulfide Bond Formation in the Bacterial Periplasm: Major Achievements and Challenges Ahead. Antioxid. Redox Signal..

[102] Rodriguez C., Nam D.H., Kruchowy E., Ge X. (2017). Efficient Antibody Assembly in E. coli Periplasm by Disulfide Bond Folding Factor Co-expression and Culture Optimization. Appl. Biochem. Biotechnol..

[103] de Marco A. (2009). Strategies for successful recombinant expression of disulfide bond-dependent proteins in Escherichia coli. Microb. Cell Fact..

[104] Seo M.J., Jeong K.J., Leysath C.E., Ellington A.D., Iverson B.L., Georgiou G. (2009). Engineering antibody fragments to fold in the absence of disulfide bonds. Protein Sci..

[105] Malherbe G., Humphreys D.P., Davé E. (2019). A robust fractionation method for protein subcellular localization studies in *Escherichia coli*. BioTechniques.

[106] Johnson B.H., Hecht M.H. (1994). Recombinant Proteins Can Be Isolated from E. coli Cells by Repeated Cycles of Freezing and Thawing. Nat. Biotechnol..

[107] Ghamghami E., Abri Aghdam M., Tohidkia M.R., Ahmadikhah A., Khanmohammadi M., Mehdipour T., Mokhtarzadeh A., Bradaran B. (2020). Optimization of Tris/EDTA/Sucrose (TES) periplasmic extraction for the recovery of functional scFv antibodies. AMB Expr..

[108] Gallegos K.M., Conrady D.G., Karve S.S., Gunasekera T.S., Herr A.B., Weiss A.A. (2012). Shiga Toxin Binding to Glycolipids and Glycans. PLoS ONE.

[109] St Hilaire P.M., Boyd M.K., Toone E.J. (1994). Interaction of the Shiga-like Toxin Type 1 B-Subunit with Its Carbohydrate Receptor. Biochemistry.

[110] Hexham J.M., Dudas D., Hugo R., Thompson J., King V., Dowling C., Neville D.M., Digan M.E., Lake P. (2001). Influence of relative binding affinity on efficacy in a panel of anti-CD3 scFv immunotoxins. Mol. Immunol..

[111] Adair J.R., Athwal D.S., Bodmer M.W., Bright S.M., Collins A.M., Pulito V.L., Rao P.E., Reedman R., Rothermel A.L., Xu D. (1994). Humanization of the murine anti-human CD3 monoclonal antibody OKT3. Hum. Antibodies Hybrid..

[112] Philipp N., Kazerani M., Nicholls A., Vick B., Wulf J., Straub T., Scheurer M., Muth A., Hänel G., Nixdorf D. (2022). T-cell exhaustion induced by continuous bispecific molecule exposure is ameliorated by treatment-free intervals. Blood.

[113] Dopfer E.P., Hartl F.A., Oberg H.H., Siegers G.M., Yousefi O.S., Kock S., Fiala G.J., Garcillàn B., Sandstrom A., Alarcón B. (2014). The CD3 Conformational Change in the γδ T Cell Receptor Is Not Triggered by Antigens but Can Be Enforced to Enhance Tumor Killing. Cell Rep..

[114] Guha P., Heatherton K.R., O’Connell K.P., Alexander I.S., Katz S.C. (2022). Assessing the Future of Solid Tumor Immunotherapy. Biomedicines.

[115] Scott E.M., Duffy M.R., Freedman J.D., Fisher K.D., Seymour L.W. (2018). Solid Tumor Immunotherapy with T Cell Engager-Armed Oncolytic Viruses. Macromol. Biosci..

[116] Hao M., Hou S., Li W., Li K., Xue L., Hu Q., Zhu L., Chen Y., Sun H., Ju C. (2020). Combination of metabolic intervention and T cell therapy enhances solid tumor immunotherapy. Sci. Transl. Med..

[117] Vierboom M.P.M., Bos G.M.J., Ooms M., Offringa R., Melief C.J.M. (2000). Cyclophosphamide enhances anti-tumor effect of wild-type p53-specific CTL. Int. J. Cancer.

[118] Ebrahimnejad P., Dinarvand R., Sajadi A., Jaafari M.R., Nomani A.R., Azizi E., Rad-Malekshahi M., Atyabi F. (2010). Preparation and in vitro evaluation of actively targetable nanoparticles for SN-38 delivery against HT-29 cell lines. Nanomed. Nanotechnol. Biol. Med..

[119] Johannes L., Goud B. (1998). Surfing on a retrograde wave: How does Shiga toxin reach the endoplasmic reticulum?. Trends Cell Biol..

[120] Kim J.H., Lingwood C.A., Williams D.B., Furuya W., Manolson M.F., Grinstein S. (1996). Dynamic measurement of the pH of the Golgi complex in living cells using retrograde transport of the verotoxin receptor. J. Cell Biol..

[121] Sandvig K., Garred Ø., Prydz K., Kozlov J.V., Hansen S.H., van Deurs B. (1992). Retrograde transport of endocytosed Shiga toxin to the endoplasmic reticulum. Nature.

[122] Sandvig K., Bergan J., Dyve A.B., Skotland T., Torgersen M.L. (2010). Endocytosis and retrograde transport of Shiga toxin. Toxicon.

[123] Lingwood C.A. (1999). Verotoxin/Globotriaosyl Ceramide Recognition: Angiopathy, Angiogenesis and Antineoplasia. Biosci. Rep..

[124] Lingwood C.A. (1994). Verotoxin-Binding in Human Renal Sections. Nephron.

[125] Furukawa K., Yokoyama K., Sato T., Wiels J., Hirayama Y., Ohta M., Furukawa K. (2002). Expression of the Gb3/CD77 Synthase Gene in Megakaryoblastic Leukemia Cells: Implication in the Sensitivity to Verotoxins. J. Biol. Chem..

[126] Nunes M.A., Vieira F.L. (1975). Negative potential level in the outer layer of the toad skin. J. Membr. Biol..

[127] Kontermann R.E. (2005). Recombinant bispecific antibodies for cancer therapy. Acta Pharmacol. Sin..

[128] Huhalov A., Chester K.A. (2004). Engineered single chain antibody fragments for radioimmunotherapy. Q. J. Nucl. Med. Mol. Imaging.

[129] Kipriyanov S.M., Moldenhauer G., Schuhmacher J., Cochlovius B., Von Der Lieth C.-W., Matys E.R., Little M. (1999). Bispecific tandem diabody for tumor therapy with improved antigen binding and pharmacokinetics. J. Mol. Biol..

[130] Klinger M., Brandl C., Zugmaier G., Hijazi Y., Bargou R.C., Topp M.S., Gökbuget N., Neumann S., Goebeler M., Viardot A. (2012). Immunopharmacologic response of patients with B-lineage acute lymphoblastic leukemia to continuous infusion of T cell–engaging CD19/CD3-bispecific BiTE antibody blinatumomab. Blood.

[131] Binnington B., Lingwood D., Nutikka A., Lingwood C.A. (2002). Effect of Globotriaosyl Ceramide Fatty Acid-alpha-Hydroxylation on the Binding by Verotoxin 1 and Verotoxin 2. Neurochem. Res..

[132] Kiarash A., Boyd B., Lingwood C.A. (1994). Glycosphingolipid receptor function is modified by fatty acid content. Verotoxin 1 and verotoxin 2c preferentially recognize different globotriaosyl ceramide fatty acid homologues. J. Biol. Chem..

[133] Wong M., Xu G., Park D., Barboza M., Lebrilla C.B. (2018). Intact glycosphingolipidomic analysis of the cell membrane during differentiation yields extensive glycan and lipid changes. Sci. Rep..

