# Supplementary material for: A Shiga Toxin B-Subunit-Based Lectibody Boosts T Cell Cytotoxicity towards Gb3-Positive Cancer Cells
Source: Cells. 2023 Jul 20;12(14):1896. doi: 10.3390/cells12141896 (PMC10378424; doi:10.3390/cells12141896)
Supplement: Supplementary file 1 [file cells-12-01896-s001.zip › cells-2406831-supplementary.pdf]

Supplementary Material

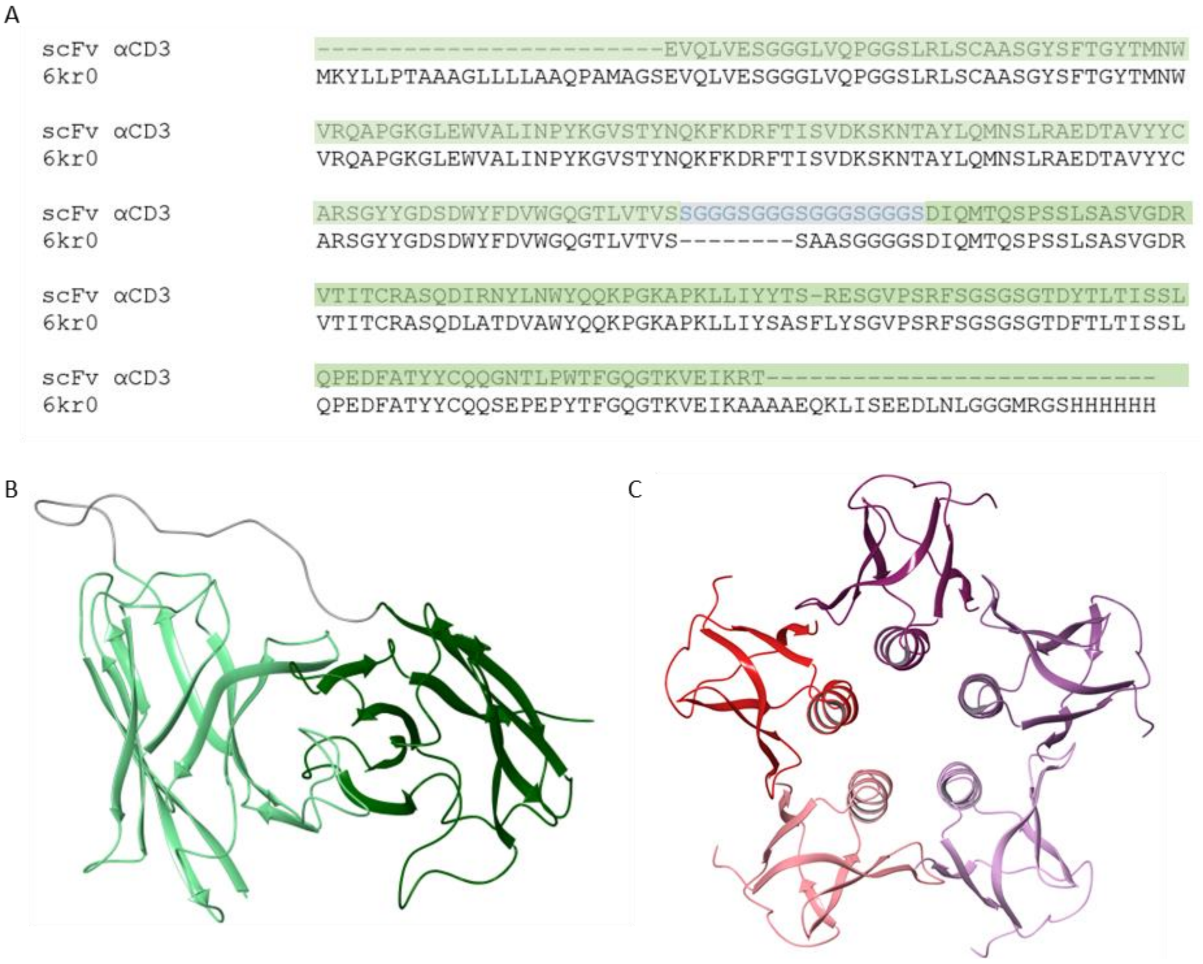

Supplementary Figure 1 Sequence alignment of the αCD3 and homology-based structures of StxB and αCD3. **A** The sequence alignment for the αCD3 scFv UCHT1 and the diabody 31 (PDB: 6kr0). The sequence of the V_H is displayed in light green, the V_L in dark green and the linker between V_H and V_L is depicted in grey. **B** The homology-based structure of αCD3 scFv UCHT1. **C** The StxB pentamer. The monomers are indicated in different colours.

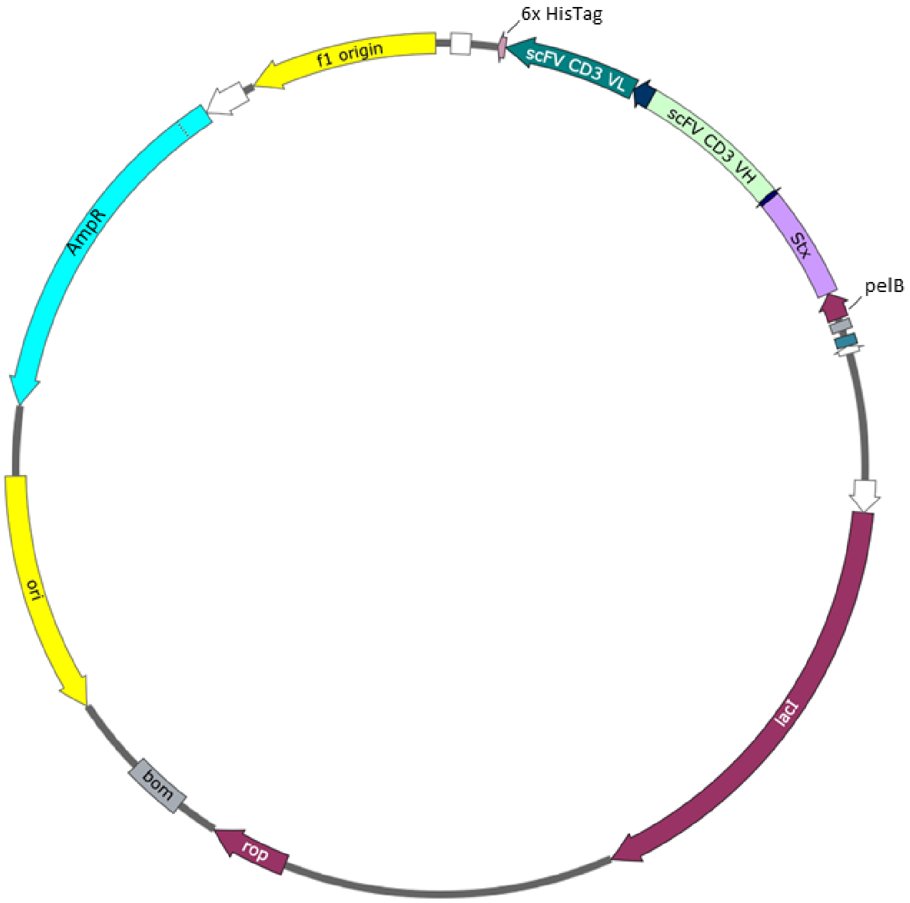

Supplementary Figure 2 Plasmid map of the pET22b StxB-scFv UCHT1 plasmid. The plasmid contains the sequence of a StxB monomer linked to the αCD3 V_H *via* a 1x GGGS linker and the αCD3 V_L *via* a 4x GGGS linker followed by a 6x HisTag used for purification. The pelB sequence included in the plasmid ensures the post-expressional translocation of the fusion protein into the periplasm. The expression of the recombinant fusion protein is under the control of the *lac* operator. The *lacI* gene encodes the *lac* repressor, it represses *lac* operator and therefore hindering gene expression. This repression can be relieved by addition of IPTG. The plasmid also includes an Ampicillin resistance (AmpR) for antibiotic selection.

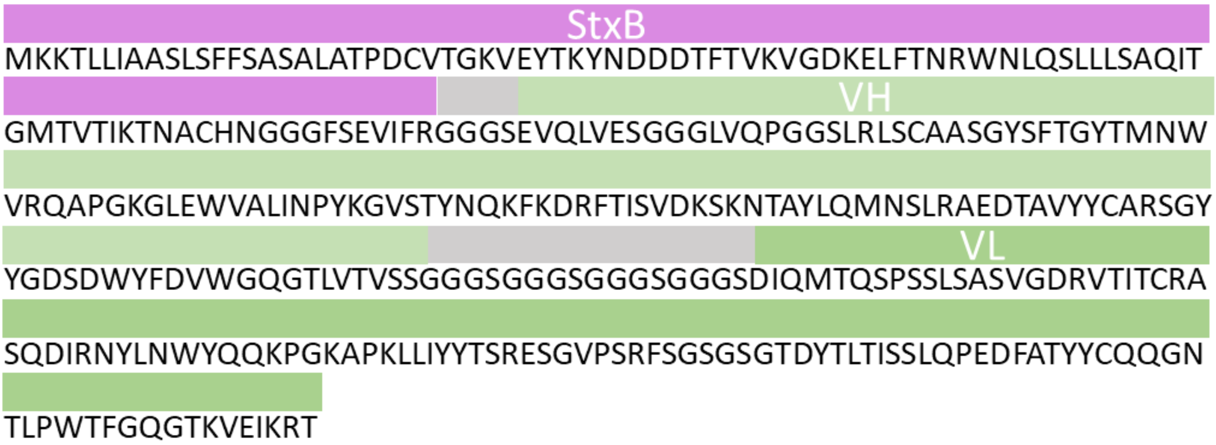

Supplementary Figure 3 Amino acid sequence of the StxB-scFv UCHT1 lectibody. The Sequence of StxB (purple) is linked to the heavy chain (VH, light green) of the scFv UCHT1via a short 1x GGGS linker (grey). The VH is followed by the light chain of the scFv UCHT1 (VL, dark green). The chains are connected via a 4x GGGS linker.

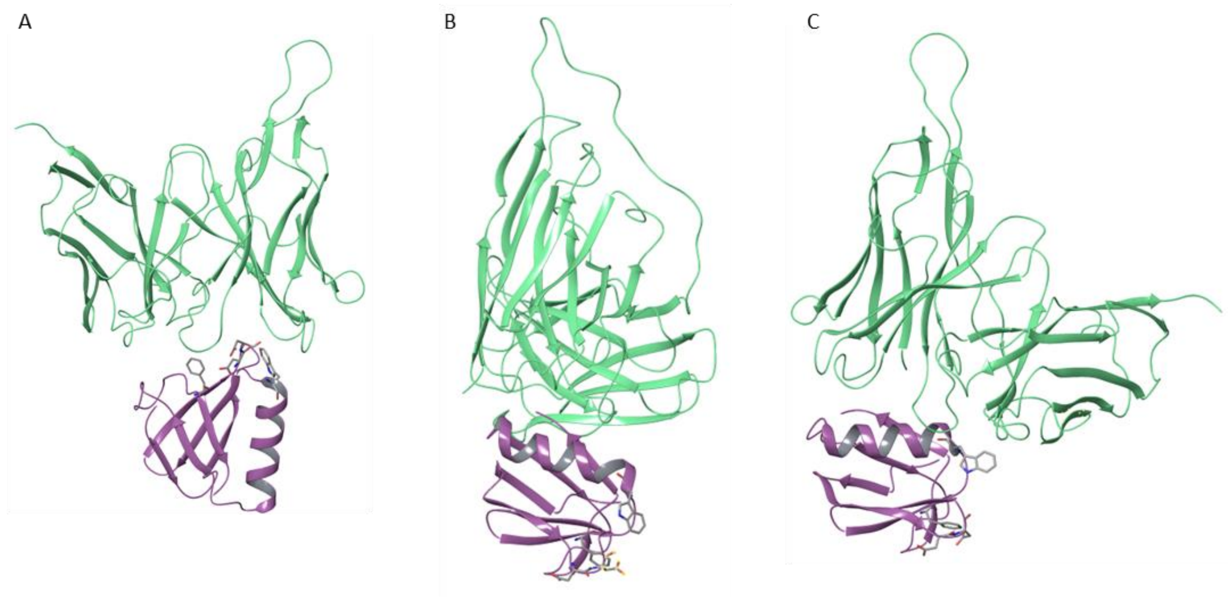

Supplementary Figure 4 A The most populated docking pose, which would require a linker length of ~30 Å. The protein-protein interactions block the Gb3 binding sites. **B, C** Two differing docking poses, which would require a shorter linker length of <12 Å. The protein-protein interactions do not block the Gb3 binding site and hide the hydrophobic region of the StxB monomer from interactions with water. Important residues for Gb3 binding are shown by sticks. Color coding: StxB – violet, scFv UCHT1 – green.

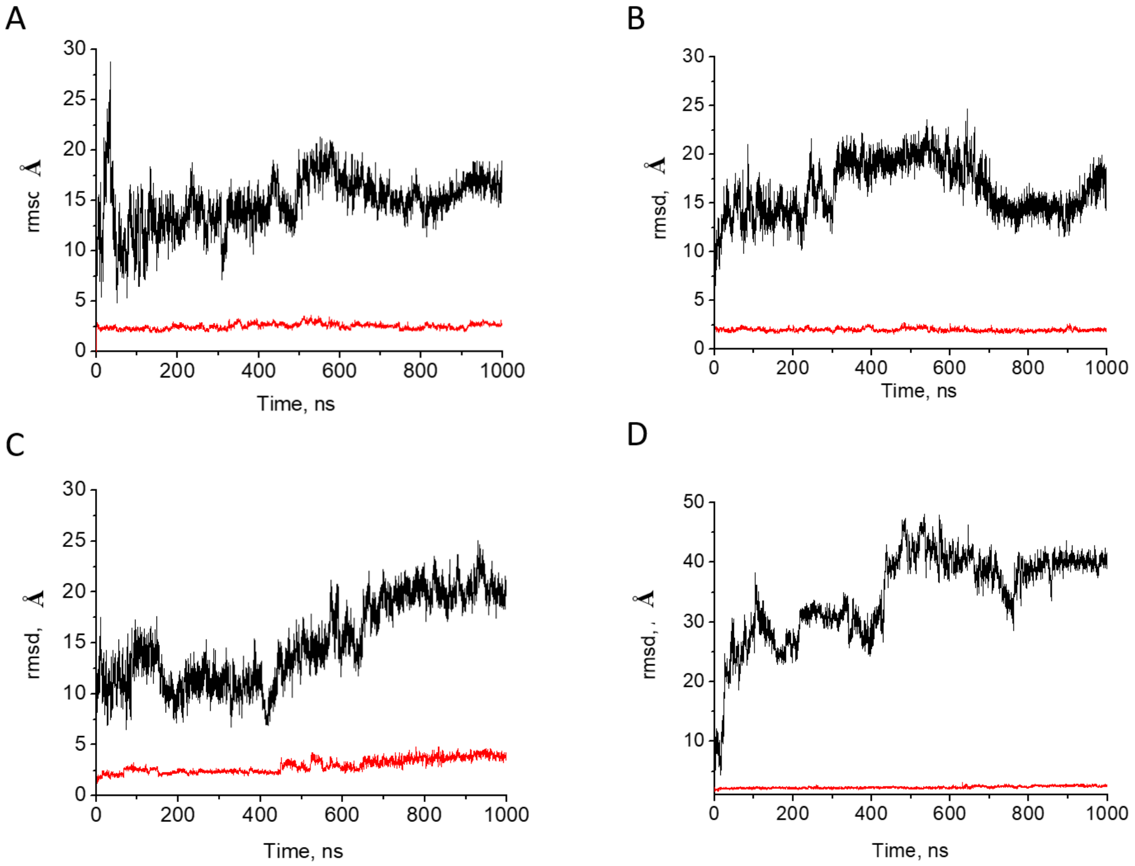

Supplementary Figure 5 Root-mean-square deviation of StxB (red) and scFv UCHT1 (black) in the course of two trajectories of StxB-scFv UCHT1 lectibody monomer (A, B) and one trajectory of StxB-scFv UCHT1 dimer (C, D), calculated on Cα atoms. Centring is done on the StxB module.

Table 1. Per-residue contacts between StxB and scFv UCHT1 in the course of two trajectories of StxB - scFv UCHT1 lectibody monomer, derived from two docking models, and lectibody dimer, derived from the equilibrated monomer model.

Residue	Mean distance, nm	SD, nm	Residue	Mean distance, nm	SD, nm	Binding energy*, kcal/mol
Monomer 1st trajectory						
ASN35	0.1947	0.02057	GLU265	0.18223	0.01687	-43.25 ± 9.48
THR48	0.19663	0.026	GLU74	0.21509	0.04766	
ARG33	0.20374	0.06656	TYR175	0.22149	0.02463	
MET48	0.22978	0.01858	TYR174	0.23819	0.01949	
THR46	0.24086	0.01901	VAL75	0.24291	0.02235	
LEU39	0.24858	0.02767	ARG264	0.24326	0.02708	
ILE67	0.24876	0.0364	TYR100	0.24517	0.02223	
SER42	0.24879	0.02349	TYR105	0.27838	0.04555	
SER38	0.25263	0.02999	GLY267	0.30107	0.07635	
ILE45	0.26891	0.03128	GLY99	0.32638	0.10263	
TRP34	0.29796	0.08362	SER266	0.33749	0.06618	
THR6	0.30047	0.05592	ARG171	0.37361	0.08896	
VAL50	0.30775	0.04597	SER270	0.38623	0.18169	
THR51	0.35186	0.05238	SER98	0.41382	0.11634	
GLU65	0.36203	0.16031	PRO269	0.42123	0.16265	
LEU36	0.40921	0.06491	VAL268	0.43642	0.0853	
VAL66	0.44673	0.10566	SER263	0.48776	0.09672	
GLY47	0.47479	0.0328	ASP183	0.49542	0.06105	
LEU41	0.48739	0.05269	GLY176	0.49711	0.04633	
Monomer 2nd trajectory						
ARG33	0.18246	0.01857	GLU265	0.18718	0.04254	-36.82 ± 5.83
THR49	0.20461	0.01681	GLY104	0.21884	0.02652	
SER42	0.21944	0.02698	TYR174	0.22043	0.01755	
SER38	0.22325	0.01868	SER101	0.2231	0.0157	
THR46	0.22348	0.01524	TYR175	0.23297	0.01767	
MET48	0.23366	0.02129	TYR105	0.23799	0.02236	
TRP34	0.23416	0.01616	GLY267	0.24828	0.02533	

LEU41	0.25345	0.02982	GLY176	0.25904	0.02358	
ILE45	0.25816	0.0282	GLY173	0.26963	0.03721	
ASN35	0.25832	0.02867	TYR127	0.27589	0.0341	
THR51	0.26186	0.06979	TYR260	0.27606	0.03145	
LEU39	0.26816	0.03711	THR103	0.27924	0.0454	
THR6	0.27019	0.06659	TYR100	0.28	0.04967	
GLN37	0.2774	0.05579	ASP177	0.28075	0.03898	
VAL50	0.31162	0.03351	SER266	0.29259	0.05219	
ILE67	0.33666	0.05918	ASP183	0.42177	0.07155	
TYR14	0.38275	0.147	ARG171	0.43873	0.08119	
PHE20	0.38826	0.15574	ARG264	0.45083	0.12316	
GLY7	0.49229	0.07482	VAL268	0.49751	0.04223	
Monomer StxB(A) – scFv(A)						
THR49	0.20256	0.04519	GLY99	0.19667	0.03712	-29.94 ± 9.23
ASN35	0.21335	0.0269	GLY267	0.21355	0.02698	
SER38	0.23282	0.04059	VAL75	0.23057	0.01715	
THR46	0.23337	0.02137	TYR260	0.23288	0.03944	
ARG33	0.23513	0.0658	TYR100	0.23926	0.01974	
MET48	0.23875	0.02153	TYR174	0.24389	0.02878	
LEU39	0.24804	0.0288	GLU265	0.24636	0.02977	
ILE45	0.25602	0.0262	ARG171	0.25318	0.02143	
SER42	0.27421	0.0575	VAL268	0.2591	0.07147	
LEU36	0.29824	0.0673	SER266	0.26029	0.03606	
LYS8	0.31691	0.10267	VAL184	0.26524	0.04034	
GLY47	0.32514	0.05031	TYR175	0.27295	0.057	
PHE68	0.33639	0.07587	LEU77	0.32847	0.0859	
ILE67	0.33794	0.10474	PRO269	0.33215	0.07835	
LEU41	0.46489	0.0551	ARG264	0.34438	0.09234	
			GLN76	0.35306	0.07724	
			SER270	0.37065	0.11533	
			SER101	0.43615	0.03922	
			SER98	0.44592	0.04701	
			ASP183	0.49323	0.0641	

			GLU74	0.49587	0.09038	
Monomer StxB(B) – scFv(A)						
ILE45	0.23293	0.01642	TYR174	0.2304	0.0205	
SER42	0.23376	0.03633	TYR173	0.23732	0.01798	
THR46	0.24295	0.02184	ARG263	0.24454	0.05307	
LEU41	0.25362	0.0379	TYR260	0.25653	0.03482	
SER38	0.29706	0.09472	TYR242	0.29295	0.05467	
			GLY175	0.45292	0.0449	
			TYR259	0.47036	0.05962	

*the binding energy was calculated at MMGBSA level as follows: $E_{binding} = E_{complex} – E_{StxB} - E_{scFv}$

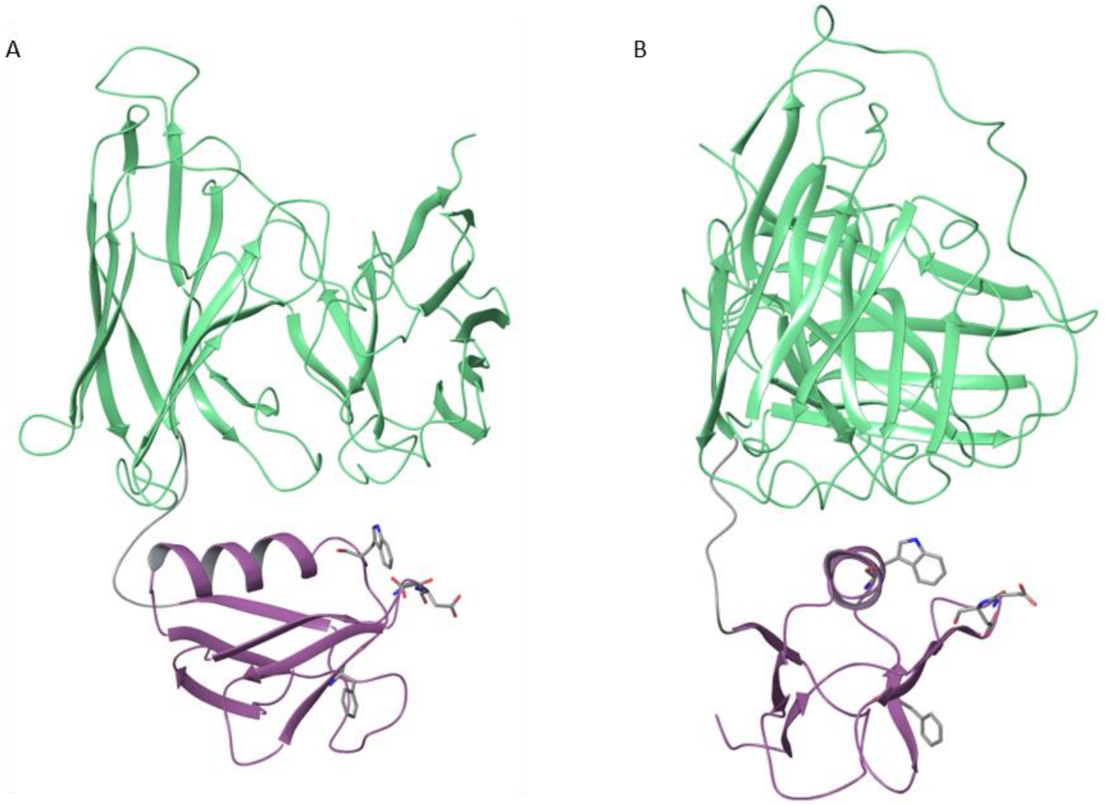

Supplementary Figure 6 Molecular dynamics trajectory of the StxB-scFv UCHT1 lectibody. **A** Front and **B** side view of StxB-scFv UCHT1 fusion protein equilibrated during MD trajectory. Important residues for Gb3 binding are shown by sticks. Color coding: StxB – violet, scFv UCHT1 – green, linker connecting StxB and scFv – grey.

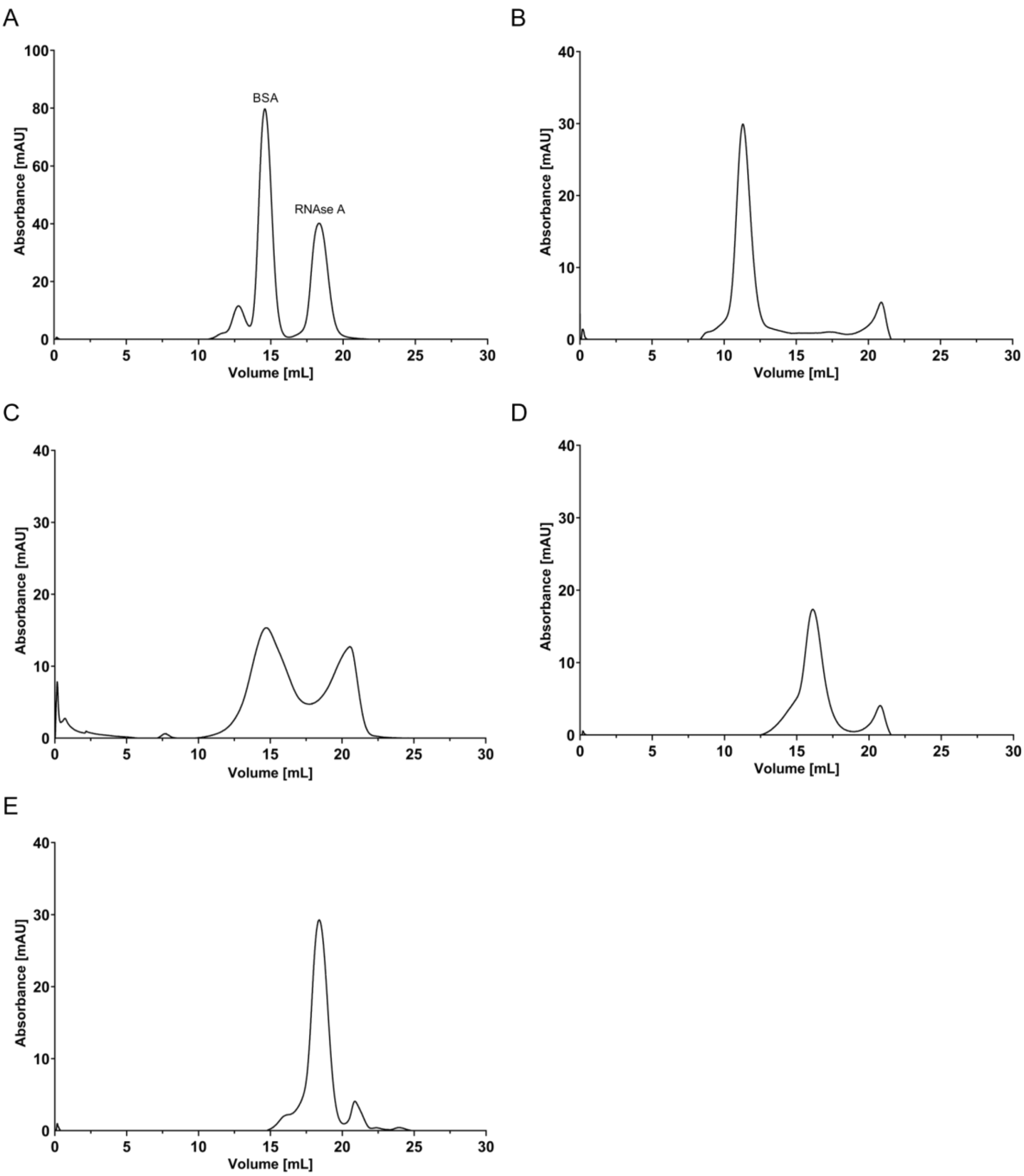

Supplementary Figure 7 Standard curve for the Superdex 200 10/300 GL column and histograms for each SEC peak for size confirmation. **A** Proteins of known size (BSA ~ 66 kDa, RNAseA ~ 13.7 kDa) were loaded onto the column to determine the elution volume for proteins of that size. BSA was eluted at 14.5 mL and RNAseA at 18 mL. When knowing the elution volume of set proteins, the elution volume of the protein of interest, in our case the StxB-scFv UCHT1 lectibody can be estimated. Elution curves for **B** peak 1, **C** peak 2, **D** peak 3 and **E** peak 4 are displayed to estimate protein size. **B** Peak 1 was eluted at ~ 11mL which according to the standard curve corresponds to ~ 440 kDa. **C** Peak 2 was eluted at ~ 14 mL, giving a size estimate of ~ 70 kDa. **D** Peak 3 was eluted at ~ 15 mL giving it a size of ~ 36 kDa. **E** Peak 4 was eluted at 18 mL making the estimated size 13.7 kDa.

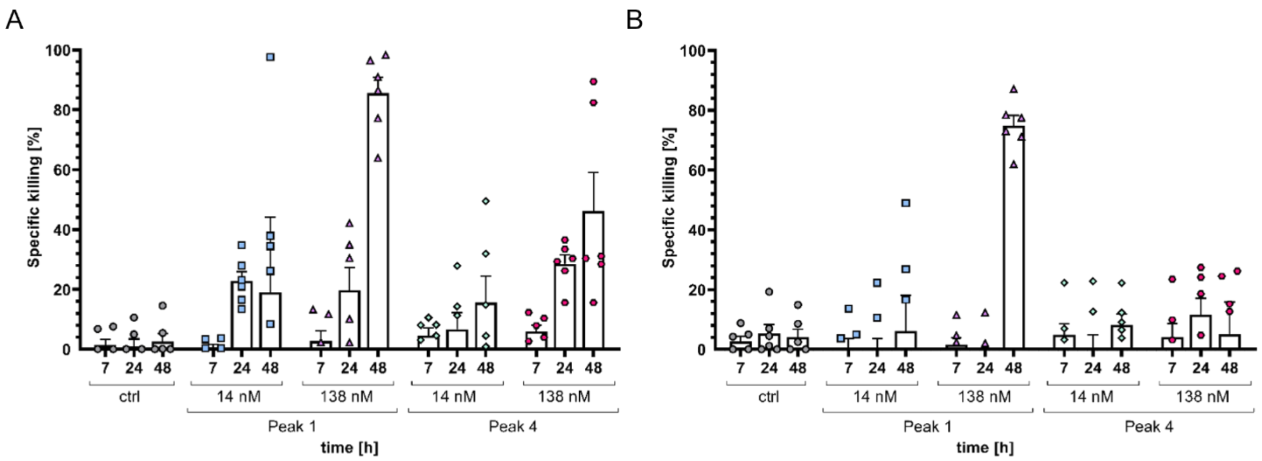

Supplementary Figure 8 Killing activity of PBMCs induced by peak 1 and 4. **A** With Gb3+ Ramos and **B** with Gb3- Namalwa cells. To assess the cytotoxic activity of T cells induced by peaks 1 and 4, target and effector cells were incubated in a ratio of 1:5. The cells were co-incubated with 14 and 138 nM of lectibody obtained from peak 1 and 4. Peak 1 induces high T cell cytotoxicity at high concentrations when co-incubated with Ramos cells but also with Namalwa. Peak 4 induces what seems dose-dependent T cell cytotoxicity at high concentration but still exhibits basal amount of killing of Namalwa cells.

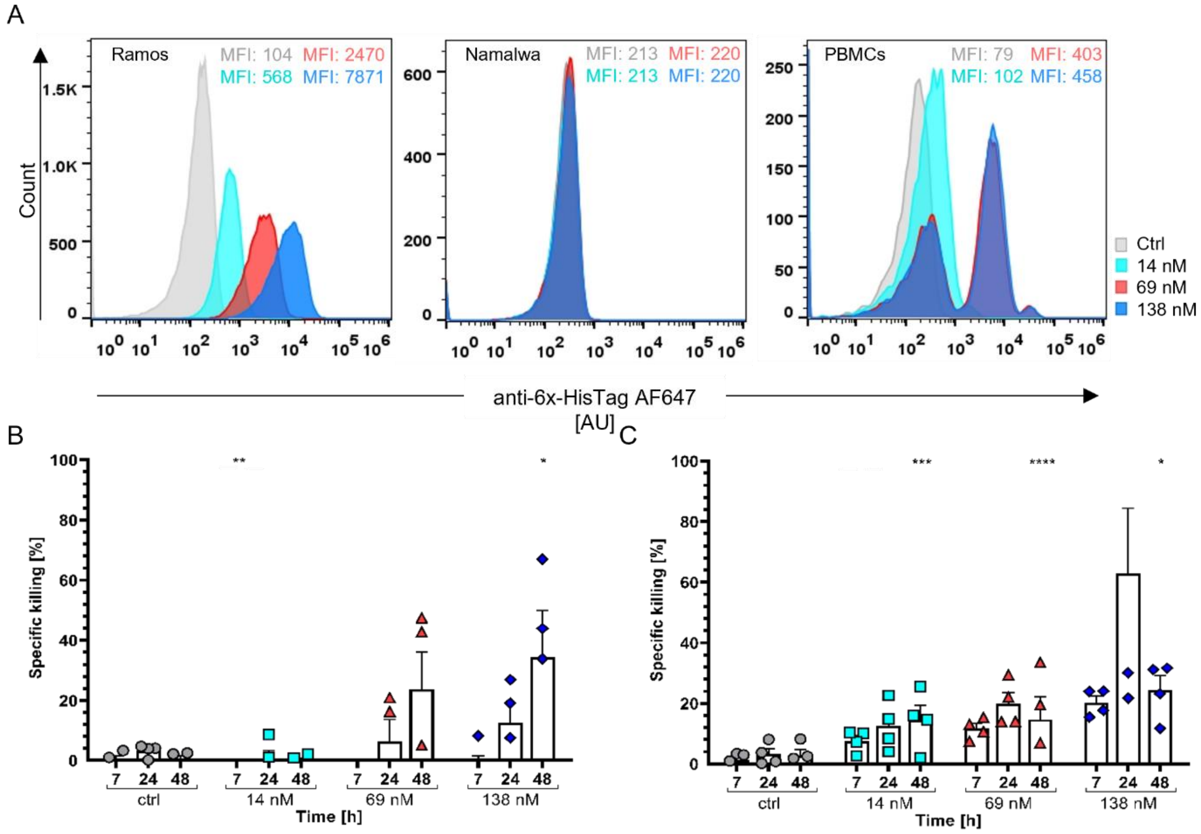

Supplementary Figure 9 Binding of monomeric StxB-scFv UCHT1 lectibody and induced T cell cytotoxicity to Burkitt's lymphoma-derived cells. **A** Representative flow cytometry histograms of Ramos cells, Namalwa cells and PBMCs incubated with the lectibody. Cells were treated with different concentrations of lectibody and stained with a fluorescent anti-6x-HisTag AF647 antibody to detect StxB-scFv UCHT1 presence at the cell surface. The fluorescence intensity is measured in arbitrary units [AU]. A dose-dependent increase in binding to Gb3 at the surface of treated cells is visible for Gb3+ Ramos, but not for Gb3- Namalwa cells. The lectibody also bound dose-dependently to the CD3 receptors on PBMCs. Mean fluorescence intensity (MFI) values depicted for every concentration. Cytotoxicity assays with **B** Ramos cells or **C** Namalwa cells were performed to assess StxB-scFv UCHT1-mediated T cell cytotoxicity. Cells were incubated with PBMCs in an effector to target (E:T) ratio of 5:1 for a period of 48 h. The specific killing activity [%] of PBMCs was measured. The data is shown as the mean ± SEM (n=4) of four separate experiments. All experiments were performed in triplicates. **B** Killing of target cells was observed for higher concentrations after 24 h of co-incubation, while at 48 h, the specific killing reached up to 32% for 138 nM StxB-scFv UCHT1. **C** There was also killing of Namalwa cells in the presence of PBMCs and StxB-scFv UCHT1 reaching around 20% for all concentrations. At 24 h with the highest concentration, there is an increase of killing activity which reduces at 48 h again to 25%, so it seems to be an outlier.

For statistical analysis, the mean of each concentration was compared to the mean of the control (ctrl) at each timepoint. $n = 4$; P-values < 0.5 were considered significant. * $p \leq 0.05$; ** $p \leq 0.01$; *** $p \leq 0.001$; **** $p \leq 0.0001$

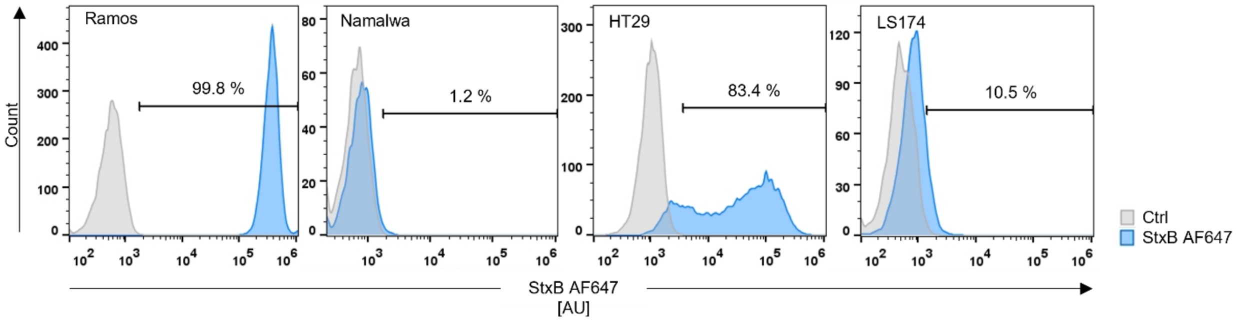

Supplementary Figure 10 StxB binding to target cell lines. Target cell lines were incubated with 8 nM StxB labelled with AF647 for 10 min at 4°C. The Burkitt's lymphoma cell line Ramos exhibited a high amount of binding (99.8%), whereas Namalwa showed only 1.2% binding of StxB. The amount of StxB binding to the colon adenocarcinoma cell line HT29 was also high (83.4%). The LS174 cells exhibited increased StxB binding compared to the Namalwa cells with 10.5%.

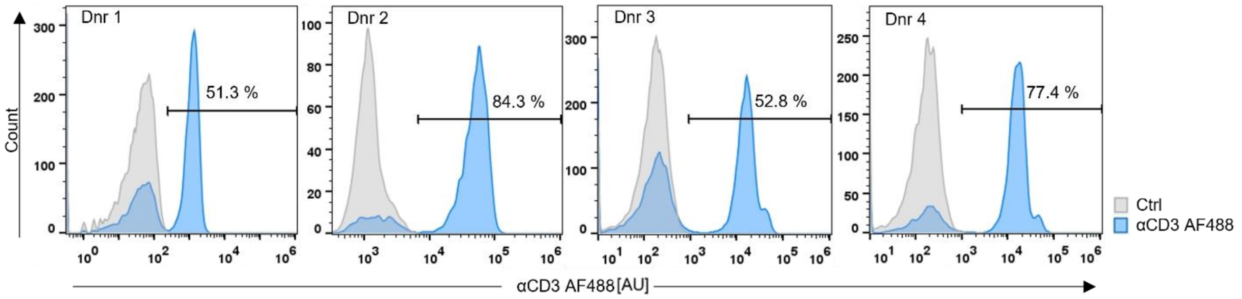

Supplementary Figure 11 αCD3 AF647 antibody binding to PBMCs. The PBMCs were isolated from four different donors (Dnr). They were incubated with αCD3 AF488 antibody for 20 min at 4°C in a dilution of 1:1000. The data showed that every donor has different amounts of T cells (CD3 positive) ranging from ~50% to 85%.

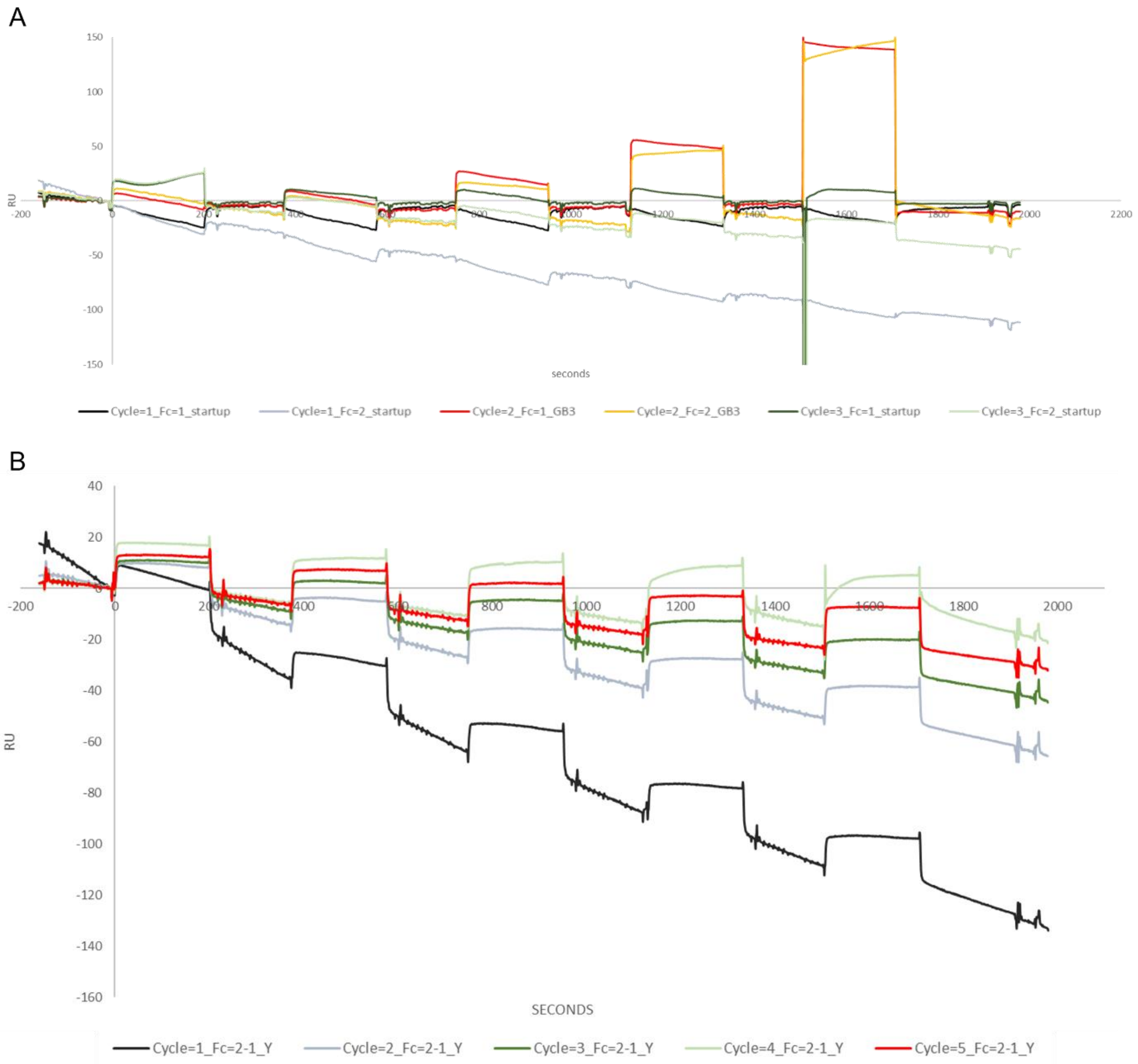

Supplementary Figure 12 Representative data from SPR measurements of **A** monomeric lectibody contained in peak 3 and **B** dimeric lectibody contained in peak 2. **A** The StxB-scFv UCHT1 lectibody was immobilized on the CM5 chip at 3800 RU (relative response). Then, a range of different Gb3 trisaccharide concentrations (5000, 1667, 555.5, 185.2 µM) was run over the surface. As the baseline went down, it indicates that the StxB-scFv UCHT1 lectibody may be unhooking from the chip as it goes. There is as much signal on the FC1 canal as on FC2 control one. After subtracting the baseline, the signals were too weak to be measured. Shown here is one exemplary run. This experiment was repeated with higher concentrations of Gb3 up to 10000 µM with similar negative result. **B** The dimeric StxB-scFv UCHT1 lectibody was therefore immobilized on the chip using amine coupling at 5877 RU. Gb3 trisaccharide was run over the surface in the same concentrations as in **A**. The baseline decreased again and there was non-specific signal and no visible interaction under these manipulation conditions.
